# Towards the bridging of molecular genetics data across Xenopus species

**DOI:** 10.1186/s12864-016-2440-9

**Published:** 2016-03-01

**Authors:** Gonzalo Riadi, Francisco Ossandón, Juan Larraín, Francisco Melo

**Affiliations:** Departamento de Genética Molecular y Microbiología, Facultad de Ciencias Biológicas, Pontificia Universidad Católica de Chile, Santiago, Chile; Centro de Bioinformática y Simulación Molecular, Facultad de Ingeniería, Universidad de Talca, Talca, Chile; Fundación Ciencia y Vida, Universidad Andrés Bello, Santiago, Chile; Center for Aging and Regeneration and Millennium Nucleus in Regenerative Biology, Santiago, Chile

**Keywords:** Xenopus, Laevis, Tropicalis, Assembly, Coarse-grained, Alignment, Map, Synteny, Genome, Sequences

## Abstract

**Background:**

The clawed African frog *Xenopus laevis* has been one of the main vertebrate models for studies in developmental biology. However, for genetic studies, *Xenopus tropicalis* has been the experimental model of choice because it shorter life cycle and due to a more tractable genome that does not result from genome duplication as in the case of *X. laevis*. Today, although still organized in a large number of scaffolds, nearly 85 % of *X. tropicalis* and 89 % of *X. laevis* genomes have been sequenced. There is expectation for a comparative physical map that can be used as a Rosetta Stone between *X. laevis* genetic studies and *X. tropicalis* genomic research.

**Results:**

In this work, we have mapped using coarse-grained alignment the 18 chromosomes of *X. laevis*, release 9.1, on the 10 reference scaffolds representing the haploid genome of *X. tropicalis*, release 9.0. After validating the mapping with theoretical data, and estimating reference averages of genome sequence identity, 37 to 44 % between the two species, we have carried out a synteny analysis for 2,112 orthologous genes. We found that 99.6 % of genes are in the same organization.

**Conclusions:**

Taken together, our results make possible to establish the correspondence between 62 and 65.5 % of both genomes, percentage of identity, synteny and automatic annotation of transcripts of both species, providing a new and more comprehensive tool for comparative analysis of these two species, by allowing to bridge molecular genetics data among them.

**Electronic supplementary material:**

The online version of this article (doi:10.1186/s12864-016-2440-9) contains supplementary material, which is available to authorized users.

## Background

African clawed frogs comprise more than twenty species of frogs native to Sub-Saharan Africa [1]. The most studied species in this genus are *Xenopus laevis* and more recently *Xenopus tropicalis*. Xenopus species have been an important model in cell biology, development, genetics and genomics. These species are an attractive model in these areas based on the ability to study embryos at all developmental stages, the presence of large eggs in abundant quantities throughout the year and the remarkable regenerative capacity in the tadpole. Xenopus research has set key principles in gene regulation and signal transduction, embryonic induction, morphogenesis and patterning as well as cell cycle regulation [2].

Historically, *X. laevis* has been considered one of the main animal models for developmental, cell, electrophysiology and biomedical studies [3–5]. However, this species presents a challenge for genomics analyses and genetics due to the *allotetraploid* nature of its genome and its long life cycle. The haploid genome of *X. laevis* has been sequenced to 89.21 % and consists of 18 chromosomes and 3.1Gbp (3.1x10^9^ bp). Current assembly of the *X. laevis* genome consists in 402,501 scaffolds in the Xenbase release 9.1 (XLA9.1) [6]. This release includes the identification of L (Long) and S (Short) chromosomes from the new nomenclature by Matsuda et. al. [7].

The *X. laevis* transcriptome counts with 45,099 primary transcript sequences. The annotation of the transcripts, in the current release, include the identification of the genes known to be duplicated, that belong to chromosomes L and S [8]. One limitation of *X. laevis*, however, has been the lack of systematic genetic studies to complement molecular and cell biology investigations. Work with the closely related diploid frog *X. tropicalis* has attempted to address this limitation [9].

*X. tropicalis* (also called *Silurana tropicalis*) is a diploid organism with 20 chromosomes and a 1.7Gbp long haploid genome. Currently, 84.81 % of the genome has been sequenced, consisting of 6,823 scaffolds in Xenbase release 9.0 (XTR9.0). The first and longest 10 scaffolds correspond to 74.88 % of contiguous sequences of the 10 haploid chromosomes in the *X. tropicalis* genome. This organism has 26,550 transcript sequences (XTR9.0). The easy molecular tractability of genomic features of *X. tropicalis* [9] has allowed integration of some genetic, biochemical, phenotypic and evolutionary data [10–14] in these two species. However, correspondence is not always expected between genomic data in *X. tropicalis* and the duplicated and divergent genome of *X. laevis* [15]. In the case there is correspondence, establishing it at a genome level is required. This cannot be done without a physical map between both genomes.

No comprehensive comparative analyses using genomic sequencing mapping have been conducted for *X. laevis* and *X. tropicalis* [16]. Aiming at facilitating such analysis, we have set out to build a comparative coarse-grained physical map between these two species. To this end, we aligned the 18 chromosomes from *X. laevis* assembly XLA9.1 to the 10 chromosomes from *X. tropicalis* assembly XTR9.0 and estimated percentage of sequence identity, repetitions, inversions and synteny of mapped genes between the two species. Finally, we validated the map theoretically through the synteny of Maximal Unique Matches (MUMs). As a whole, our results convey the suitability of this newly assembled map for comparative studies between these two species, bridging a long-standing gap for the integration of biochemical, genetic and genomics data in Xenopus.

## Results

**Fig. 1:**
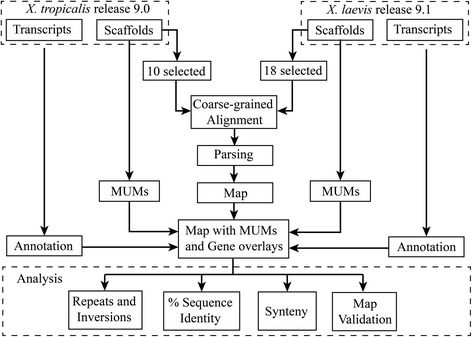
A chart summarizing the workflow from the two *Xenopus* assemblies to the map and the analyses

In this work we have performed a comparative analysis between the two frog genomes after mapping by a coarse-grain alignment method the chromosome sequences of *X. laevis* on the chromosome sequences from *X. tropicalis* and semi automatic annotation of their transcripts (Fig. 1) to complement the map information. The analyses include a validation of the map, estimations of percentage of sequence identity, repetitions, inversions and synteny between the two genomes.

### The map

As *X. laevis* genome is around 1.8 times the length of *X. tropicalis* genome, 1.8 is also the expected rate of added lengths of the blocks aligned between the two species. This rate depends on the alignment drop-off score, X, chosen. A resulting rate larger than 1.8 suggests a loose alignment. On the other hand, a resulting rate smaller than 1.8 suggests a strict alignment. The drop-off score X = 35,000 rendered an average alignment length rate of 1.77, which is close to the expected rate (Table 1). However, the rate between the lengths of the chromosomes from *X. laevis* respect to *X. tropicalis* is 2.15, larger than expected.

**Table 1:** Summary of the coarse-grained map between 18 XLA9.1 chromosomes (L and S) on 10 XTR9.0 chromosomes. The length units are in blocks. Each block corresponds to a sequence of length 5 Kbp. Xtr (*X. tropicalis*); Xla (*X. laevis*); Chr (Chromosome)

Xtr Chr	Xtr blocks length	Xtr blocks aligned	Xtr coverage	Number Xla Chr aligned	Total Xla Chr length	Xla Chr blocks aligned	Xla Chr coverage	Alignment rate	Length rate
1	38980	30325	0.778	2	79980	69269	0.70	1.85	2.05
2	34048	24941	0.733	2	68224	53657	0.66	1.82	2.00
3	27458	21199	0.772	2	52947	47149	0.73	1.82	1.93
4	26703	20991	0.786	2	53082	46342	0.73	1.84	1.99
5	29320	20599	0.703	2	59210	43481	0.62	1.79	2.02
6	27032	19736	0.730	2	56700	42203	0.64	1.83	2.10
7	23449	14512	0.619	2	43314	30723	0.59	1.77	1.85
8	23530	17076	0.726	2	43836	34028	0.64	1.64	1.86
9	16091	11011	0.684	2^a^	44503	24682	0.44	1.77	2.77
10	7993	5124	0.641	2^a^	44503	10047	0.18	1.57	5.57
Totals	254604	185514	0.73	18	546299	401581	0.74	1.77	2.15

^a^In *X. laevis*, chromosomes 9 and 10 from *X. tropicalis* become fused and duplicated. They were named Chr9_10L and Chr9_10S chromosomes in XLA9.1. The same set was aligned to *X. tropicalis* chromosome 9 and chromosome 10

**Fig. 2:**
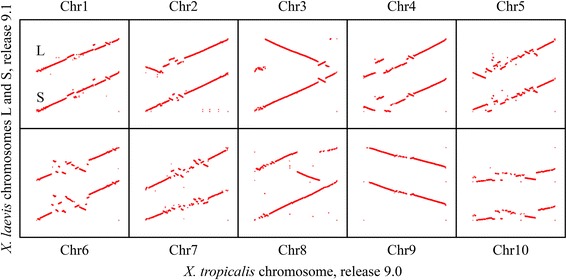
Dotplot alignments of each XLA9.1 L and S chromosomes (y axis) to each XTR9.0 chromosome (x axis). A red dot represents a block alignment between *X. laevis* and *X. tropicalis* chromosomes. The alignments are not at the same scale

A coarse-grained dotplot alignment between *X. laevis* scaffolds and each *X. tropicalis* chromosome scaffold shows graphically part of the information in Table 1 (Fig. 2). Although the alignments seem to be contiguous, overall 27.1 % of *X. tropicalis* chromosomes did not align to *X. laevis* chromosomes. In supplement to this figure, the proportion of *X. tropicalis* chromosomes covered by *X. laevis* was 72.9 % (Table 1). This proportion, combined with the completion of 84.81 % of the *X. tropicalis* genome (Additional file 1), results that 61.8 % of *X. tropicalis* whole genome is actually aligned by *X. laevis* blocks. A similar coverage of 65.5 % was obtained for *X. laevis* chromosomes (Table 1).

### Conservation between *X. tropicalis* and *X. laevis*

**Fig. 3:**
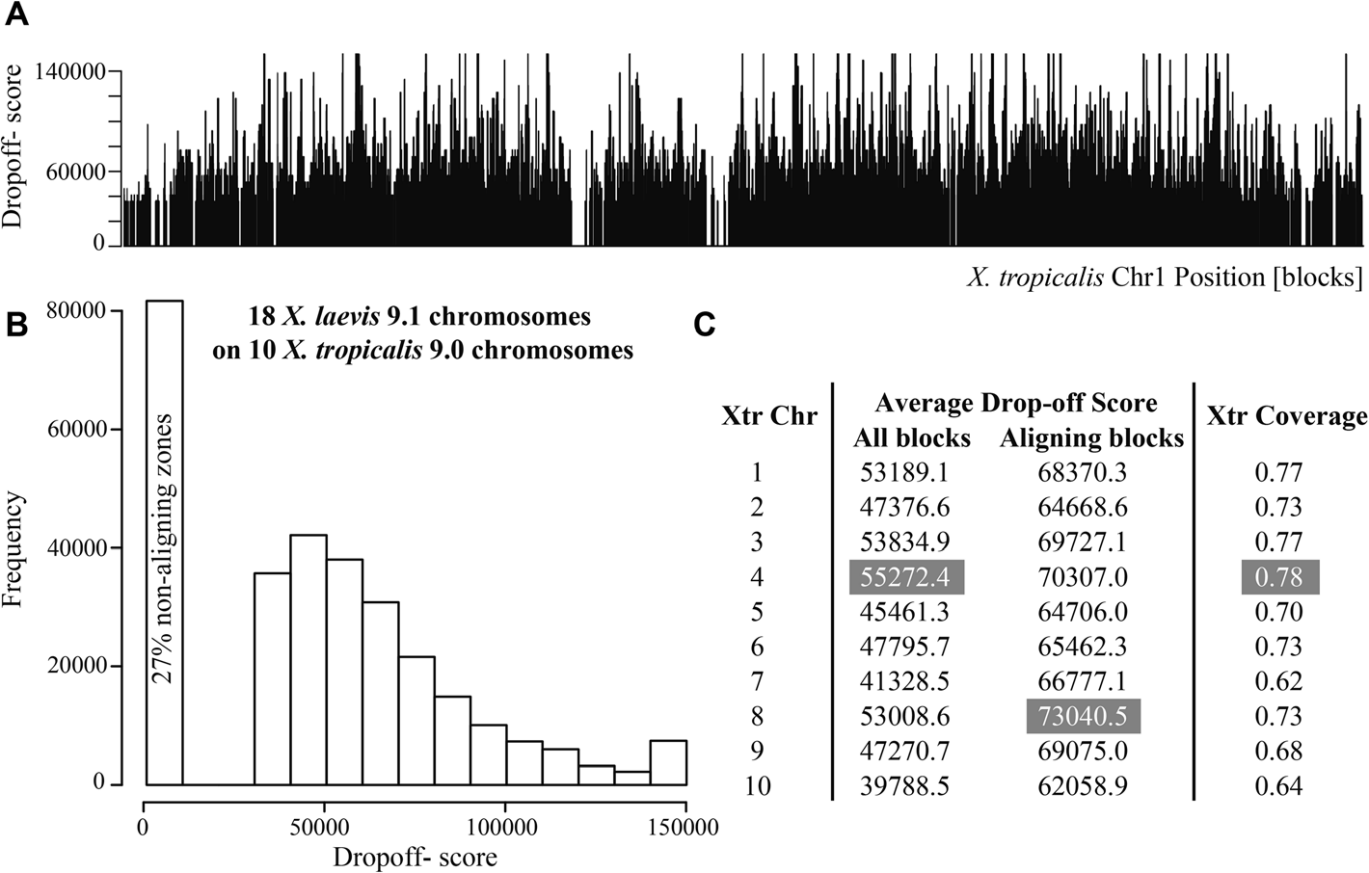
Maximum drop-off scores chart of *X. laevis* blocks on *X. tropicalis*. **a** bar-plot of the maximum drop-off score, X, per block position for chromosome 1. **b** Histogram of drop-off scores. **c** Table with the average maximum drop-off scores per chromosome calculated using all blocks, only the aligning blocks, and the coverage of the aligning blocks on *X. tropicalis*. The cells in grey show the most conserved chromosomes according each column

As the resulting alignment depends on the drop-off value used, we aligned all *X. laevis* scaffolds against all *X. tropicalis* chromosomes at 24 increasing drop-off score values (35,000-150,000 with a pace of 5,000) (Fig. 3). The block positions that appear with no conservation are either not aligned or have a score lower than 35,000, in which case cannot be distinguished from chance. The maximum drop-off score at which a pair of blocks can be aligned correlates directly with percentage of sequence identity between aligned sequences. However, as the variance of the percentage of sequence identity per drop-off score value is significant, the percentage of sequence identity cannot be reliably predicted from the drop-off score. In spite of this, the maximum drop-off score at which a pair of blocks is aligned can be used as a measure of conservation. From each chromosome, a histogram of maximum drop-off scores or conservation scores was generated and the coverage of alignment for each drop-off was calculated. The average maximum Cgaln drop-off score between the aligning zones of the genomes is 67,703.32 (Fig. 3). Possibly, the histogram of maximum drop-off scores shows a larger than expected proportion of conserved blocks with score of 150,000, as that bin accumulates all blocks with drop-off score 150,000 or higher. Chromosome 10 is the shortest chromosome, and the one that has the lowest average conservation (Fig. 3) and lowest alignment coverage (Table 1). In order, from highest to lowest average conservation we have *X. tropicalis* chromosomes: 4, 3, 1, 8, 6, 2, 9, 7 and 10 (averaging through all the chromosome sequence, including the non aligned regions). This chromosome conservation order changes to 8, 4, 3, 9, 1, 7, 6, 5 and 10 if the averaging only takes into account the aligning blocks.

### Repetitions and inversions

As *X. laevis* genome is the result of whole genome duplication event, it is expected that 1.8 *X. laevis* blocks will align each *X. tropicalis* block. Therefore, a block of nucleotides cannot simply be regarded as a block that happens more than once in a genome. Three particular cases have to be taken in account: a block from *X. tropicalis* that aligns to *X. laevis* is considered a repeat when (i) it is an additional block to an already-aligned first block at one particular scaffold; (ii) it belongs to a third scaffold in addition to two previous aligned scaffolds or; (iii) it is a combination of the former two cases.

In this map, a total of 11.8Mbp from *X. tropicalis* are repeated in 26.6Mbp in the *X. laevis* aligned genome (Additional file 2). Inversions are identified only for colonies, i.e., with at least two consecutive aligning blocks [24]. For colonies, a previous check on the scaffold frame is made, as in Cgaln only the best out of the 6 reading frames of each *X. laevis* scaffold is aligned. An inversion is identified when Cgaln takes the plus frame of the *X. laevis* chromosome and a colony is aligned in reverse respect to the *X. tropicalis* chromosome. Because only colonies in reverse can be identified, the inversions counted are an underestimation of the total number of existing inversions. Taking into account this limitation, we estimated at least 64.6Mbp to be inverted between the two genomes. Inversions represent 7 % and 3 % of the aligned portion of *X. tropicalis* or *X. laevis* genomes, respectively (Table 2).

**Table 2:** Summary of repetitions (repeated blocks) and inversions in the coarse-grained map between 18 XLA9.1 chromosomes on 10 XTR9.0 chromosomes. Columns 2 to 5 are sub estimates of the number of repeated blocks from each genome that align on the other genome. Columns 6 to 8 are sub estimates of inversions between the genomes

Xtr Chr	Repetitions of Xtr	Repetitions on Xla	Repetitions of Xla	Repetitions on Xtr	Inversion blocks	Inversions on Xtr	Inversions on Xla
1	0.32	0.30	0.16	0.43	1305	0.04	0.02
2	0.31	0.24	0.16	0.34	1291	0.05	0.03
3	0.31	0.26	0.18	0.41	1708	0.08	0.04
4	0.33	0.25	0.18	0.36	1420	0.07	0.04
5	0.32	0.25	0.16	0.32	1249	0.06	0.03
6	0.31	0.24	0.15	0.31	1055	0.05	0.03
7	0.31	0.25	0.17	0.35	1673	0.12	0.07
8	0.30	0.25	0.19	0.35	1428	0.08	0.05
9	0.28	0.25	0.21	0.47	1339	0.12	0.07
10	0.24	0.21	0.21	0.39	450	0.09	0.06
Totals	0.30	0.25	0.18	0.37	12918	0.07	0.03

### Validation of the map

In order to validate the map between *X. laevis* and *X. tropicalis*, we computed a set of common theoretical probes called Maximal Unique Matches (MUMs, see Methods) between the two genomes and compared their correlative order in the map. The MUMs generated were identical between species and 250 nt or longer.

The distribution of distances between the corresponding positions in the map for the MUMs gives a measure of how well the correspondence between the genomes was achieved. The generated list of MUMs has 1,140 sequences. From those, 1,092 were mapped on the ten *X. tropicalis* chromosomes and 695 were mapped on the *X. laevis* scaffolds; 673 MUMs, representing 59.0 % of the total, are common and mapped to both species. This number is less than expected as it is lower than the proportion of the *X. laevis* genome mapped. Additionally, 661, or 98.2 % of the mapped MUMs on *X. laevis* are at a distance of ≤5Kbp from the corresponding MUM in *X. tropicalis*. One block, or 5Kbp, is the resolution of the map. Therefore, we estimate that the correspondence between the two sets of scaffolds was achieved in 98.2 % of the map.

### Application of the map: Conserved synteny and gene rearrangements

To calculate conserved synteny, a set of orthologous genes between two species is required. 7,910 orthologous genes were found through bidirectional-best-hit using blastn. A subset of these, 7,218 genes, map on the *X. tropicalis* 10 chromosomes.

Out of all *X. laevis* transcripts, only 9,269 map on *X. tropicalis* chromosomes (Table 3). From these, 2,112 are orthologous genes and present in at least pairs of consecutive orthologous genes mapped in the same *X. laevis* chromosome. This set was our orthologous genes sample for synteny estimation. We found that 2,105 orthologous genes, or 99.6 % of the sample, are syntenic between the two species.

**Table 3:** Distribution of XLA9.1 transcripts according to its mapping on XTR9.0 chromosomes assembly. A transcript is considered partially aligned if only one of the blocks, either the one including the start or the stop position, is aligned. A transcript does not align on *X. tropicalis* if neither of the blocks that include start or stop positions, is aligned

Category	Number of transcripts	Percentage [%]
Mapped on Xtr	9,269	20.5
Not in mapped Xla chrs	4,493	10.0
Partially align on Xtr	6,567	14.5
Do not align on Xtr	24,770	55.0
Total	45,099	100.0

**Fig. 4:**
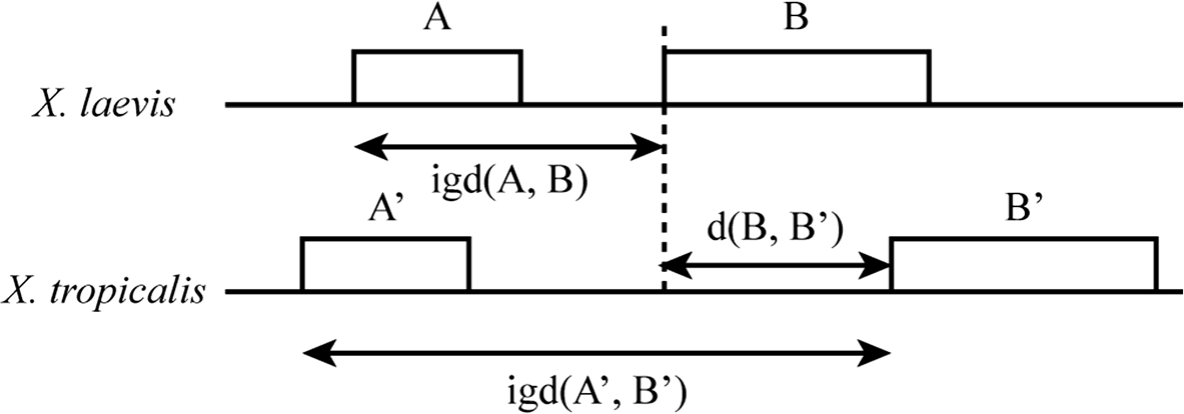
Three distances measured between the consecutive genes in *X. laevis* (XLA9.1), A and B as intergenic-distance, igd(A, B); between two consecutive genes in *X. tropicalis* (XTR9.0), A’ and B’ as igd(A’, B’) and; the distance between orthologous genes in both genomes d(B, B’)

Because the intergenic distance is one of the main determinants of order conservation [17], three distances were measured between pairs of orthologous genes (Fig. 4): 1) Distance between two consecutive genes in *X. laevis*; 2) distance between two consecutive genes in *X. tropicalis* and; 3) distance between *X. laevis* start block position projected on *X. tropicalis* and its orthologous gene start block.

The relative error of the distance between two consecutive genes in *X. laevis* respect to *X. tropicalis* was calculated with the first two distances. The mean relative error was 4.5 %. This means that regardless the absolute distance between two consecutive orthologous genes in *X. tropicalis*, the corresponding consecutive genes in *X. laevis* are, in average, ± 4.5 % of that distance apart. 71.1 % of the orthologous pairs of genes are in the corresponding block position according to the map. In the case of the distribution for the third measured distance, it was found that orthologous genes are mapped, in average 9Kbp, and that 95 % of the orthologous genes are at most 55Kbp apart. For comparison, the confidence interval of lengths, at 95 %, of Xenopus genes are between 5 and 15Kbp.

### Percentage sequence identity between the two species

Based on the calculated mapping between the two species, and to assess more precisely the sequence conservation, a random sample containing 100Mbp of matching blocks were aligned by using the global Needleman-Wunsch and local Smith-Waterman dynamic programming algorithms. The aim was to estimate, respectively, upper and lower references of the sequence identity between the two Xenopus species.

**Fig. 5:**
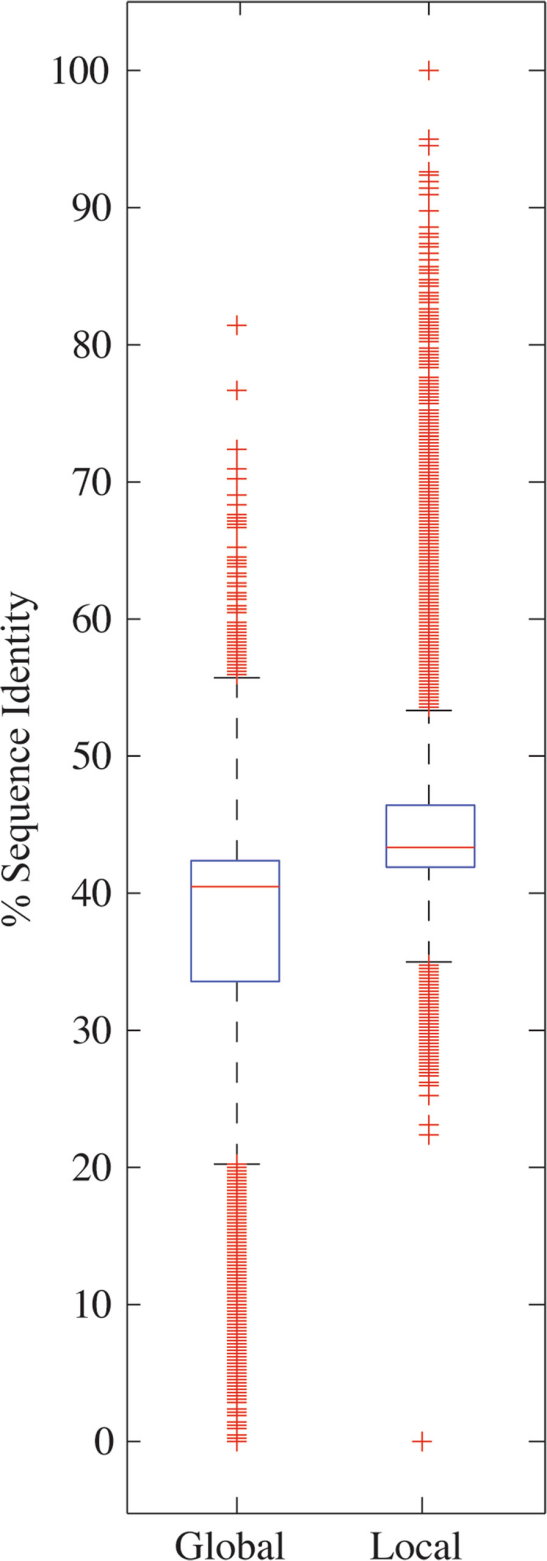
Boxplot of the global and local alignment sequence identities of the 20,000 samples of pairs of blocks from all chromosomes. The box in the boxplot concentrates 50 % of the data. The whiskers are 1.5x the length of the box. The red crosses represent outliers

For the two types of alignments, median percentage sequence identities are similar, both per chromosome and in total (Table 4). The distributions for global and local alignment overlap (Fig. 5). The medians are 40.9 and 43 %, respectively. In average, the percentage sequence identity shared by the two species ranges between 37.44, for global, and 44.08 %, for local alignments.

**Table 4:** Statistics of sequence identity between XLA9.1 and XTR9.0 genome assemblies. The sampling size of couples of aligned blocks between *X. tropicalis* and *X. laevis* was 20,000 (or 100Mbp) for all chromosomes

XtrChr	Average [%]		Median [%]		St.Dev [%]	
	Global	Local	Global	Local	Global	Local
1	36.83	45.06	40.10	43.70	9.58	7.68
2	37.24	45.56	40.40	44.00	9.37	7.55
3	37.48	45.42	40.40	43.70	8.94	6.99
4	36.79	44.92	40.60	43.40	9.63	7.45
5	37.58	43.76	41.00	43.10	8.90	7.00
6	37.95	43.71	41.53	43.10	8.48	6.01
7	37.53	44.52	40.90	43.20	8.87	7.31
8	37.00	44.88	40.60	43.50	9.69	8.23
9	38.24	42.30	41.40	42.50	8.30	5.66
10	37.76	40.69	41.00	41.90	8.45	6.22
All	37.44	44.08	40.90	43.00	9.04	7.20

## Discussion

In this work we have used *X. tropicalis* first 10 scaffolds (XTR9.0) as reference for the coarse-grained mapping of the 18 largest *X. laevis* scaffolds (XLA9.1). Using this strategy, we were not only able to map the genes and calculate the conserved synteny of orthologs between these two species but also estimate the percentage of global identity, inversions and repetitions. Taken together, this newly assembled map represents a useful tool for the integration between biochemical, physiological, genetic and genomics data between *X. laevis* and *X. tropicalis*.

### The map

The expected alignment rate is around 1.8 considering the rate of genome length between the two species. Our data show a similar alignment rate of 1.77. It was also expected the length rate between *X. laevis* respect to *X. tropicalis*, *i.e.*, the rate between the length of the scaffolds that align, to be 1.8 but rather we calculated a length rate of 2.15. It is possible that this difference either reflects evolutionary features such as genome rearrangements, translocations, deletions and fusions [18], or are associated with assembly artifacts.

The gaps in Xenopus genomes impinge on mapping and gene identification [19]. About 89.2 % of *X. tropicalis* and 84.8 % of *X. laevis* genomes were used for the mapping. If we assume that the two genomes are two random sequences of the same size, it is expected that 0.892 x 0.848 = 75.6 % of *X. tropicalis* genome actually aligns. The alignment coverages in *X. tropicalis* and *X. laevis* genomes is 61.8 and 65.5 %, respectively, lower than expected. The non-aligned blocks, or misalignments, may be due to recombination, deletion or insertion of sequences in both species [20]. Whole genome duplication is known to cause recombination and pseudogenization among other adaptive processes [21]. The rearrangements that happened in segments either smaller than 5Kbp in one single block or ≥5Kbp and ≤10Kbp combined in two consecutive blocks might not align with a score over the drop-off score in Cgaln.

### Repetitions and inversions

The meaning of the repetition figures is that 11.8Mbp from *X. tropicalis,* are aligned with 11.8Mbp in *X. laevis*, and blocks of 5Kbp in that sequences are repeated at least once in additional 26.6Mbp in the *X. laevis*.

Regarding inversions, 64.6Mbp is the estimated length between the two species. However, this is an underestimate as the inversion identification relies on the colonies aligned, and these only represent a subset of the inversions. Inversions represent 7 % of the aligned portion of *X. tropicalis* genome and 3 % of the aligned portion of *X. laevis* genome. These figures depend on the assembly quality; therefore will probably change in the next releases of Xenopus assemblies (see Previous assembly releases, below).

Inversions and repetitions are associated with evolutionary rearrangement events [22]. Each chromosome alignment (Fig. 2), assuming a correct assembly, reveals a few large rearrangements. In a few cases, for example in chromosome 6, chromosomes L and S show the same general pattern, which suggests that these rearrangements took place before the genome duplication event in the common ancestor between Xenopus species. In other cases, the differences between L and S chromosomes, for example chromosome 8, indicate a rearrangement after the genome duplication event. The alignments of L and S chromosomes against *X. tropicalis* chromosomes 9 and 10 show the fusion point in *X. laevis*. The patterns suggest that the chromosomes fusion event took place before the genome duplication event. Often, the border regions of large rearrangements contain long repetitions in the order of 10^5^ to 10^6^ bp. Additional analysis of the border regions of these hypothetical rearrangements may confirm them, further validating the assembly.

### Previous assembly releases

Assembly releases XTR8.0 and XLA7.1, available in 2014, were coarse-aligned and analyzed using the same methodology described in this work. The sequences aligned included the largest 3,169 from XLA7.1 and the largest 10 scaffolds from XTR8.0, which constitute around 80 % of each genome. The map had an overall coverage of about 50 % of both genome sequences (compare to 62–65.5 % of genome sequence coverage in this work). This suggests that new assembly releases may change alignment coverage significantly. The estimation of inversions was 58 %, largely due to the lack of contiguity of XLA7.1 assembly. Other map features, like alignment rate, repetitions, percentage of sequence identity and gene synteny estimated between the genomes, as expected, confirm the results drawn with releases XTR9.0 and XLA9.1, used in this work. Additional map validation was performed using FISH results from [16]. As the updated versions XLA9.1 and XTR9.0 were already refined by fluorescence in situ hybridization (FISH) experiments [6], such validation was not needed in this study.

## Conclusions

Overall, our results indicate that the final map aligns between 62 and 65.5 % of *X. tropicalis* and *X. laevis* total genome length despite the fact that the two species are close to be completely sequenced. The current map allowed an estimation of genome sequence identity between these species (37-44 %); the location of 9,269 genes of *X. laevis* and 20,323 genes in *X. tropicalis*, (7,218 orthologous), the automatic annotation of the transcripts of both species, and the calculation of the conserved synteny between the two frog species verifying the correspondent positions of 2,105 pairs of orthologous genes (99.6 %), making this a useful source for future comparative studies between *X. laevis* and *X. tropicalis*.

## Methods

### Scaffold sets used and selected for alignment

Both Xenopus species scaffolds sets were downloaded from Xenbase FTP site [6, 23]. After downloading the sequenced data sets (*X. laevis* 9.1 and *X. tropicalis* 9.0), we charted a superior accumulative distribution ordered by length for each organism (Additional file 1). Coarse-grained alignment is able to align a pair of large sequences, saving computational resources, by dividing the sequences into blocks of nucleotides [24]. We chose the alignment block size to be 5Kbp, because this figure represented a good compromise between the diminishing number of *X. laevis* scaffolds and the increasing of loss of information in terms of base pairs (Additional file 1). 5Kbp is also, approximately, a lower boundary for the average size of a Xenopus gene. Based on this block size definition, the longest 18 and 10 scaffolds, were selected, making up 80,93 % and 74.88 % of the haploid genomes of *X. laevis* and *X. tropicalis*, respectively (Additional file 1).

### Parameters for coarse-grained alignment

Cgaln was chosen for coarse-grained alignment [24]. In a Cgaln charted output alignment, a dot represents an alignment between two blocks of nucleotides, and is generated if the alignment score is above a given drop-off threshold, determined as X in Cgaln parameters. The minimum drop-off score X was chosen to assure that single dots were not generated by chance. This critical X value was found by generating a large number of random pairs of nucleotide sequences of 5Kbp with different known % G + C content. Each pair was then aligned at increasing drop-off score (5,000-150,000 with a pace of 5,000), to find the minimum score over which the single dot from a random alignment is not generated. The minimum drop-off score was found to be X = 35,000. This strict criterion assures that single dots generated by Cgaln have in average 43 % of global sequence identity for 5Kbp block sequences (data not shown).

### Coarse-grained mapping of *Xenopus laevis* scaffolds over *Xenopus tropicalis* reference chromosomes

Cgaln starts by dividing the sequences in blocks of user defined size. We used blocks of 5Kbp. The steps of the alignment are similar to other programs and are three: Finding High-Scoring-Pairs (HSPs), Extension and Chaining HSPs. Just as two letters have a similarity score between them, for a pair of blocks a similarity score is calculated probabilistically using the number of common k-mers found. After a first identification of similar “block seeds”, the alignment is chained and extended. As the alignment extends, the gapped blocks penalize the total score. The alignment stops the extension when the score falls below a user defined drop-off score. The default drop-off score, X, is 5000.

The output of an alignment is a file with a list of coordinate pairs, (x; y) of a dotplot, each one representing the alignment between two blocks of 5Kbp from the two species. In our case, the x-axis is the block position of the reference, *X. tropicalis*, and the y-axis is the block position of *X. laevis* scaffold. A continuous set of aligned blocks, at least two in sequence, is called colony. An alignment between two sequences may contain several colonies.

Perl scripts were written to parse the output of Cgaln and identify by chromosome position blocks of *X. laevis* scaffolds aligned in *X. tropicalis*. The scripts also identify and count repetitions and inversions.

### Validation of the map

The map was validated through the determination of the set of identical and unique subsequences of maximal length between the two sets of scaffolds: Maximal Unique Matches or MUMs. The assumption is that the corresponding MUMs in the two species genomes should align or be located at a short distance in the map. MUMs can be used to test theoretically the overall synteny between the two genomes and can be recalculated in the upcoming releases of the assemblies, to be used in map validation. The list of MUMs was generated through Vmatch (http://www.vmatch.de/). First mkvtree, part of Vmatch, was used to generate an indexed database of *X. tropicalis* scaffolds sequences with options: −v dna -allout. Then, we used vmatch command on *X. laevis* scaffold sequences, using *X. tropicalis* database, to find the MUMs over 250 nt, between the two species. For that, we used with options –mum and –l 250. Finally, we merged the MUM positions with the rest of the map using a Perl script.

### Percentage of sequence identity estimation between the two species

The percentage of sequence identity between the two species was estimated by randomly sampling 20,000 pairs of blocks of 5Kbp, 2,000 per chromosome, derived from the alignment. Global and local alignments of the pairs were carried out with EMBOSS’ Needleman & Wunsh and Smith & Waterman algorithms implementations through the command lines needle and water [25], respectively.

### Determination of a strict orthologous gene subset

*X. tropicalis* has 26,550 annotated transcripts in release XTR9.0. *X. laevis* has 45,099 annotated transcripts in release XLA9.1. In order to determine a strict orthologous subset, a bidirectional-best-hit using blastn [26] was applied to the two species sets of all transcripts. The filtering criteria were >50 % of query sequence length coverage and >60 % sequence identity in the alignment.

### Conserved synteny

There are several definitions [27] and methodologies described to calculate synteny [28]. In this work we used the conservation of similar gene orders in multiple genomic regions [29]. We estimated quantitatively the conserved synteny as the proportion of orthologous genes mapped on both species that are in the same order. The order was verified taking consecutive pairs of orthologous genes between the two species. The distance between the start blocks of the orthologous genes were recorded and, if the order was conserved in both species, it was counted as a syntenic pair. The sample size used was 2,112 because from the 7,910 orthologous genes, this was the number of genes that were accompanied by at least a second orthologous gene mapped in the same *X. laevis* chromosome.

### Annotation of transcripts

A semi automatic pipeline was used to annotate the transcripts from the two species in order to complement map information. The nucleotide sequences were translated into their 6 reading frames, and used as query in locally run BLAST against several sequence and domain databases such as TnpPred [30], CDD [31], COG [32], KOG [33], PDB [34], Pfam [35], PRK [36], SMART [37], TIGRFAMs [38], UniProt/Swiss-Prot [39]. The BLAST parameters configured include the use of low complexity sequence filtering (SEG) and discarded hits that had an e-value higher than 10^−5^ or less than 20 % of hit coverage. In the next step, the pipeline algorithm chose the best hit found for each mRNA from all the hits obtained from all the databases results. The algorithm considered the best BLAST values (e-value, score, sequence identity), but also assigned more weight to hits from better curated databases (e.g. TIGRFAMs hits weight more than UniRef90 hits), and assigned priority to informative gene product descriptions (e.g. a “glutamate decarboxylase” hit is preferred over a “hypothetical protein” hit). Finally, a table was printed with the relevant information of the annotation predictions (Additional file 3).

## Additional files

Additional file 1:
**Tables with accumulative superior distribution of scaffold lengths of** ***X. tropicalis*** **release XTR9.0 and** ***X. laevis*** **release XLA9.1 assemblies for comparison.** (PPTX 763 kb) Additional file 2:
**Map of** ***X. laevis***, **XLA9.1, 18 chromosomes on** ***X. tropicalis*** **, XTR9.0, 10 chromosomes.** (XLSX 22301 kb) Additional file 3:
**Annotation of biological function of transcripts of XLA9.1 and XTR9.0.** (ZIP 29029 kb)

## Abbreviations

Cgaln: Coarse-grain alignment
FISH: Fluorescence In Situ Hybridization
Gbp: 10^9^ base pairs
HSP: High Scoring Pairs
L: Long (chromosome from *X. laevis*)
Mbp: 10^6^ base pairs
MUM: Maximal Unique Match
S: Short (chromosome from *X. laevis*)
X: Drop-off score
XLA#.#: *Xenopus laevis* genome assembly release #.#
XTR#.#: *Xenopus tropicalis* genome assembly release #.#
