# Supplementary material for: Towards the bridging of molecular genetics data across Xenopus species
Source: BMC Genomics. 2016 Mar 1;17:161. doi: 10.1186/s12864-016-2440-9 (PMC4772642; doi:10.1186/s12864-016-2440-9)
Supplement: Additional file 1: — Tables with accumulative superior distribution of scaffold lengths of X. tropicalis release XTR9.0 and X. laevis release XLA9.1 assemblies for comparison. (PPTX 763 kb) [file 12864_2016_2440_MOESM1_ESM.pptx]

## Slide 1
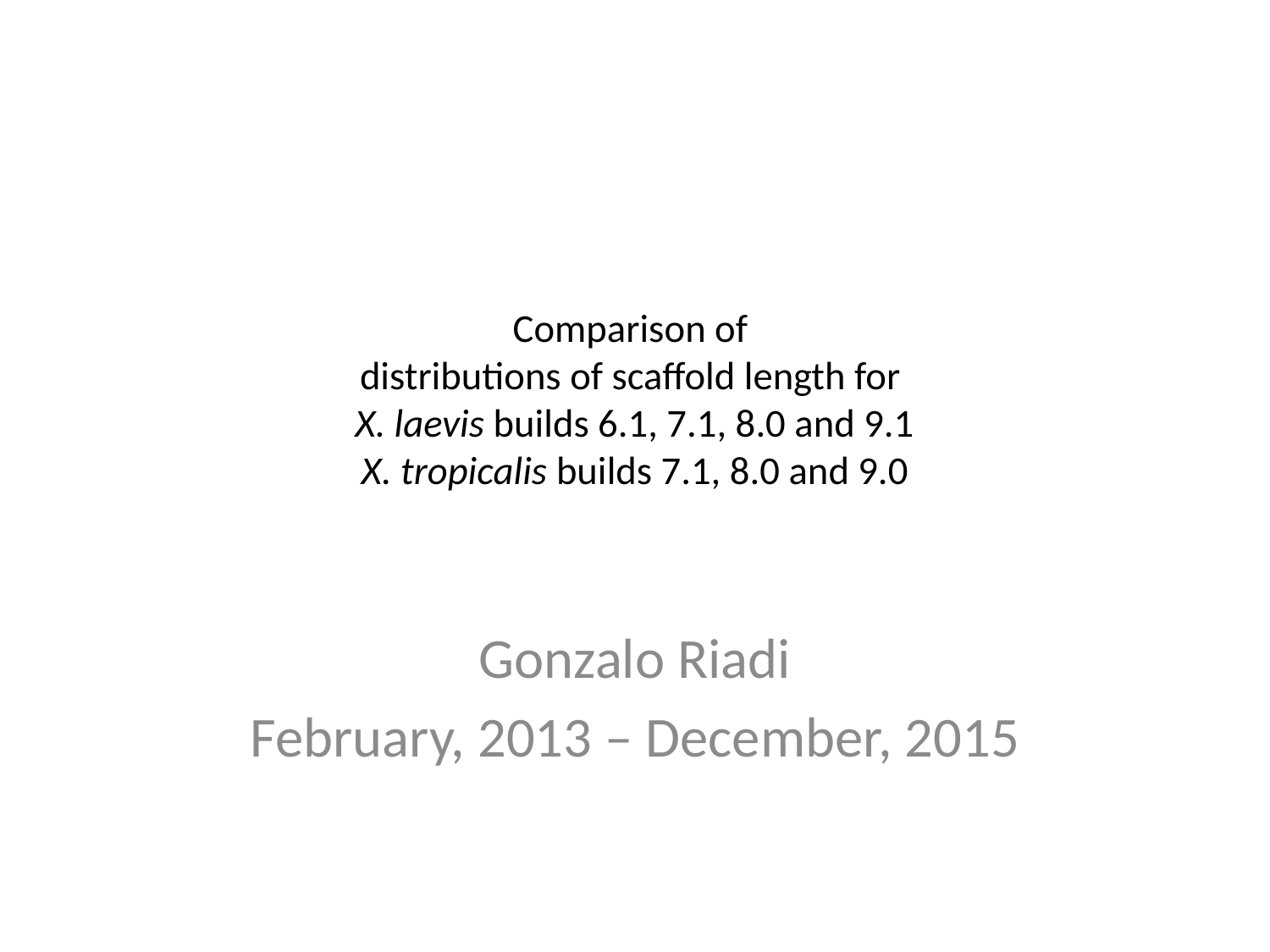

# Comparison of distributions of scaffold length for X. laevis builds 6.1, 7.1, 8.0 and 9.1 X. tropicalis builds 7.1, 8.0 and 9.0
Gonzalo Riadi
February, 2013 – December, 2015

## Slide 2
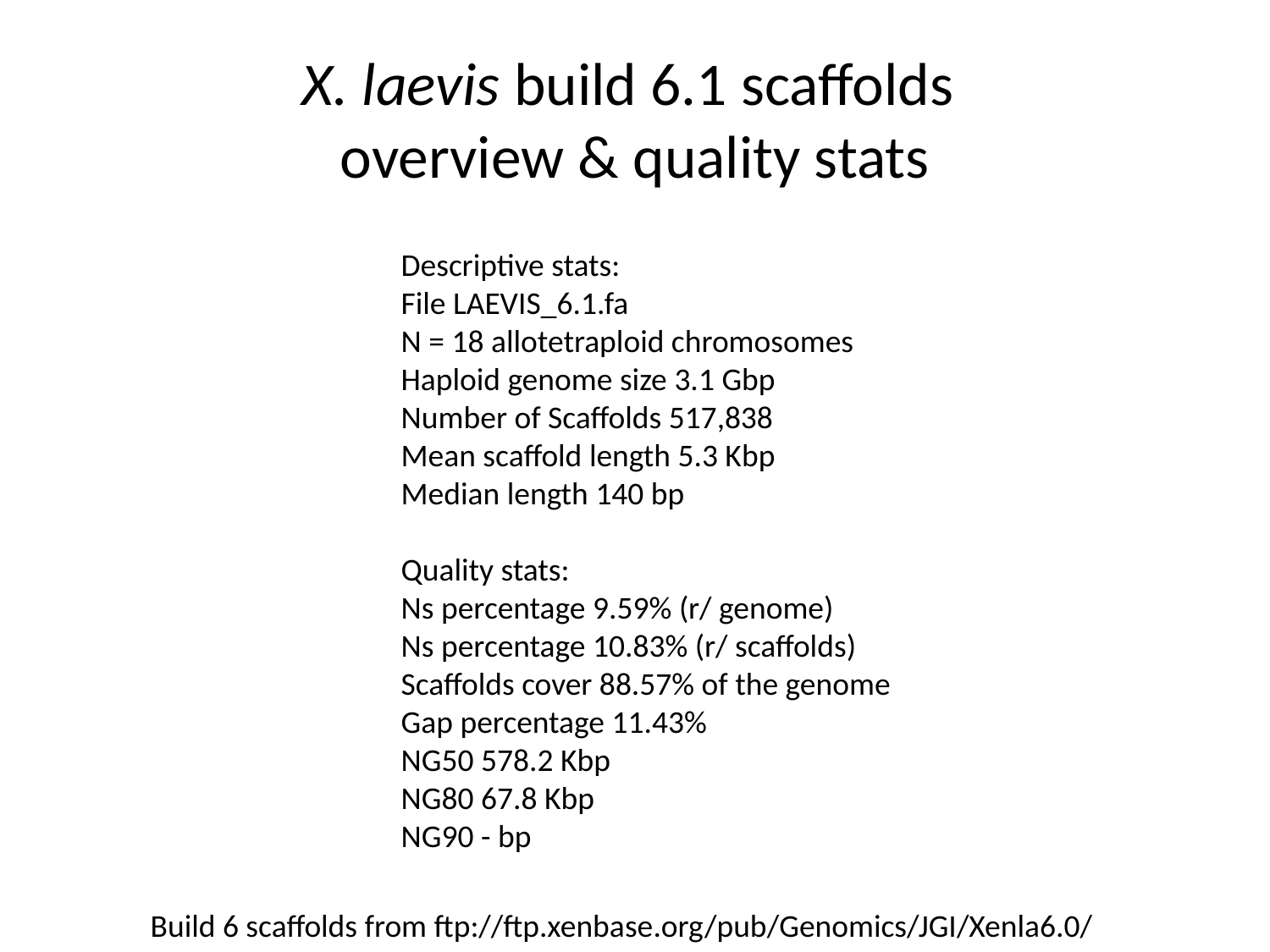

# X. laevis build 6.1 scaffolds overview & quality stats
Descriptive stats:
File LAEVIS_6.1.fa
N = 18 allotetraploid chromosomes
Haploid genome size 3.1 Gbp
Number of Scaffolds 517,838
Mean scaffold length 5.3 Kbp
Median length 140 bp
Quality stats:
Ns percentage 9.59% (r/ genome)
Ns percentage 10.83% (r/ scaffolds)
Scaffolds cover 88.57% of the genome
Gap percentage 11.43%
NG50 578.2 Kbp
NG80 67.8 Kbp
NG90 - bp
Build 6 scaffolds from ftp://ftp.xenbase.org/pub/Genomics/JGI/Xenla6.0/

## Slide 3
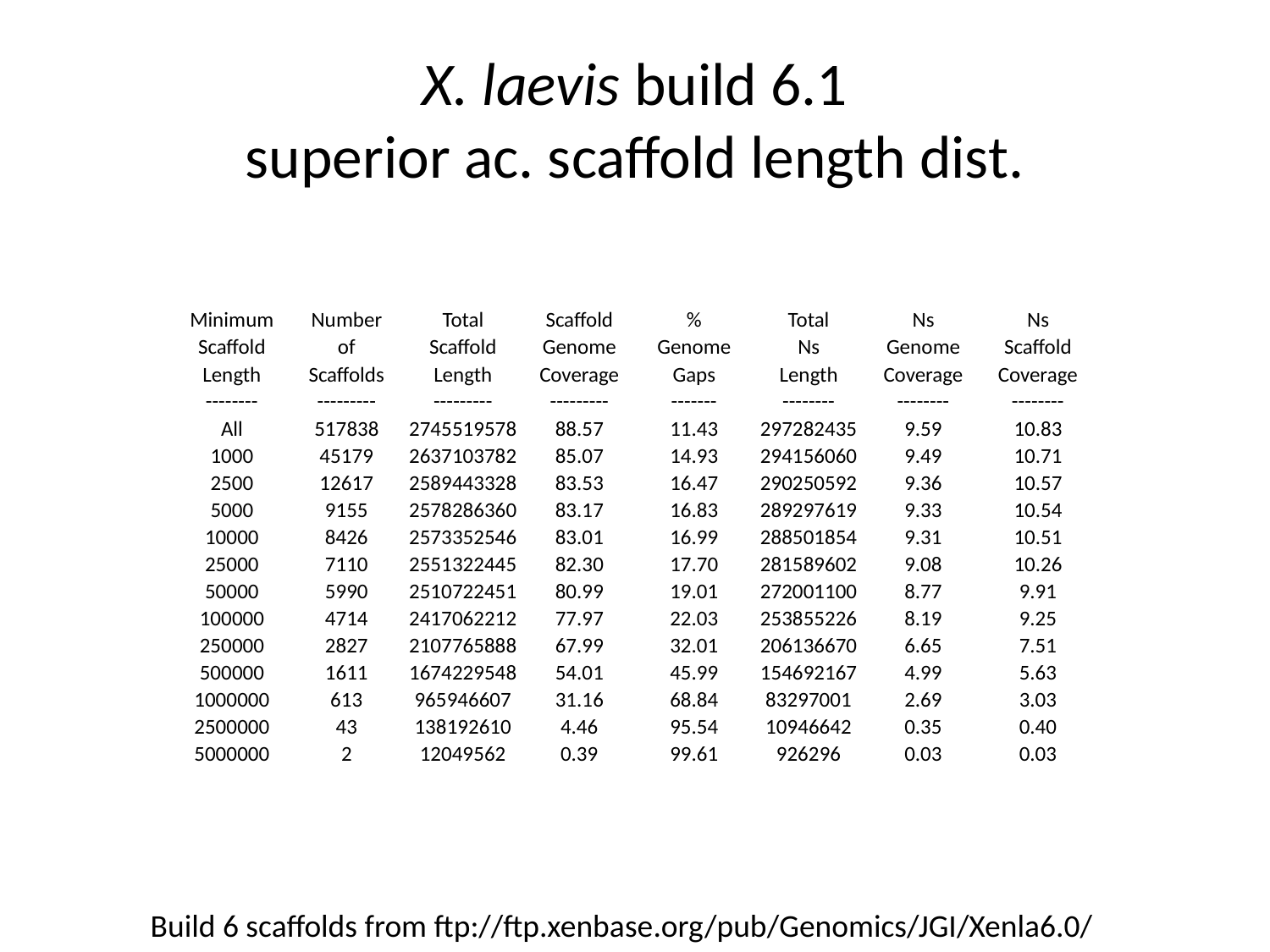

# X. laevis build 6.1 superior ac. scaffold length dist.
| Minimum | Number | Total | Scaffold | % | Total | Ns | Ns |
| --- | --- | --- | --- | --- | --- | --- | --- |
| Scaffold | of | Scaffold | Genome | Genome | Ns | Genome | Scaffold |
| Length | Scaffolds | Length | Coverage | Gaps | Length | Coverage | Coverage |
| -------- | --------- | --------- | --------- | ------- | -------- | -------- | -------- |
| All | 517838 | 2745519578 | 88.57 | 11.43 | 297282435 | 9.59 | 10.83 |
| 1000 | 45179 | 2637103782 | 85.07 | 14.93 | 294156060 | 9.49 | 10.71 |
| 2500 | 12617 | 2589443328 | 83.53 | 16.47 | 290250592 | 9.36 | 10.57 |
| 5000 | 9155 | 2578286360 | 83.17 | 16.83 | 289297619 | 9.33 | 10.54 |
| 10000 | 8426 | 2573352546 | 83.01 | 16.99 | 288501854 | 9.31 | 10.51 |
| 25000 | 7110 | 2551322445 | 82.30 | 17.70 | 281589602 | 9.08 | 10.26 |
| 50000 | 5990 | 2510722451 | 80.99 | 19.01 | 272001100 | 8.77 | 9.91 |
| 100000 | 4714 | 2417062212 | 77.97 | 22.03 | 253855226 | 8.19 | 9.25 |
| 250000 | 2827 | 2107765888 | 67.99 | 32.01 | 206136670 | 6.65 | 7.51 |
| 500000 | 1611 | 1674229548 | 54.01 | 45.99 | 154692167 | 4.99 | 5.63 |
| 1000000 | 613 | 965946607 | 31.16 | 68.84 | 83297001 | 2.69 | 3.03 |
| 2500000 | 43 | 138192610 | 4.46 | 95.54 | 10946642 | 0.35 | 0.40 |
| 5000000 | 2 | 12049562 | 0.39 | 99.61 | 926296 | 0.03 | 0.03 |
Build 6 scaffolds from ftp://ftp.xenbase.org/pub/Genomics/JGI/Xenla6.0/

## Slide 4
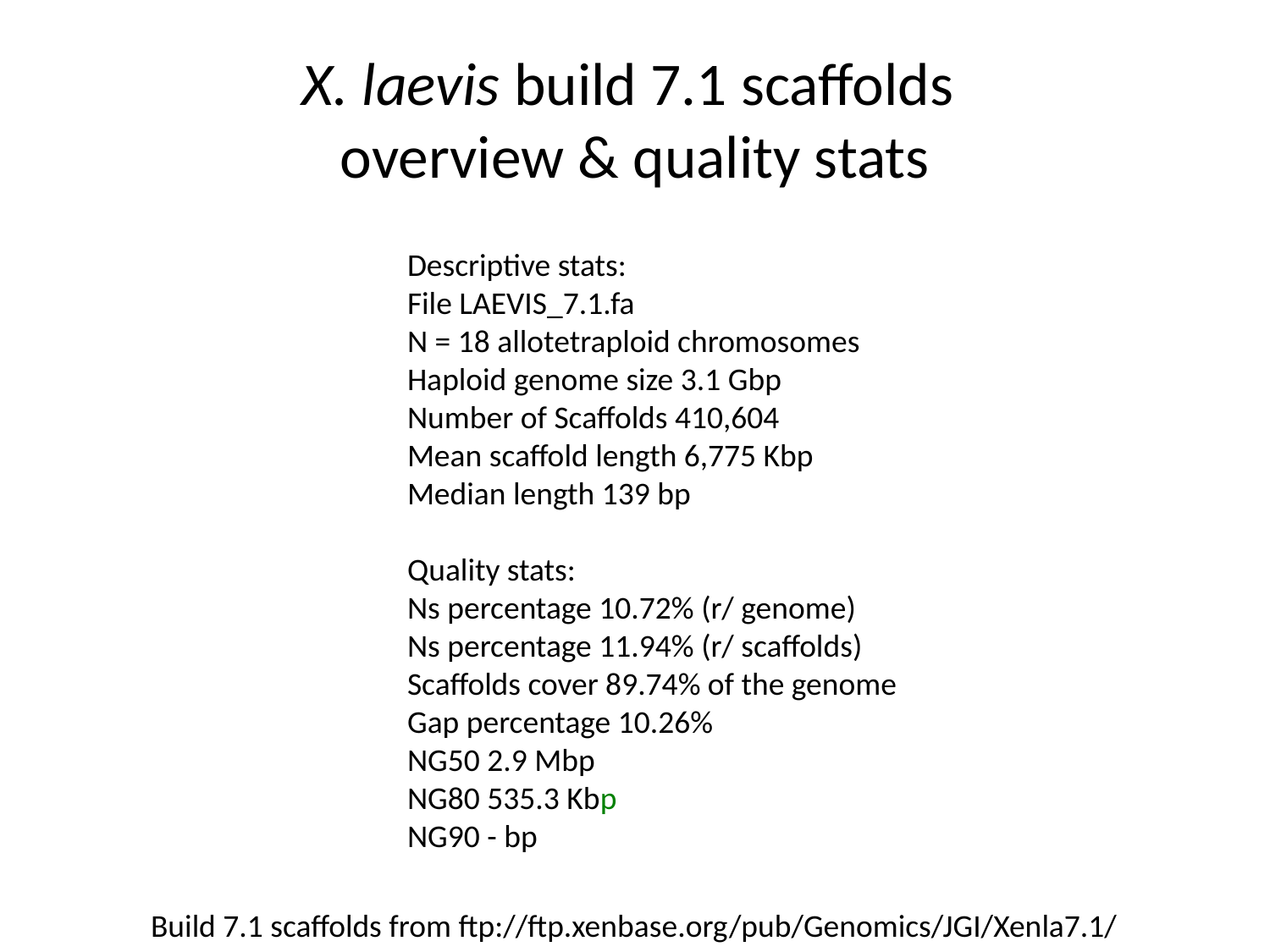

# X. laevis build 7.1 scaffolds overview & quality stats
Descriptive stats:
File LAEVIS_7.1.fa
N = 18 allotetraploid chromosomes
Haploid genome size 3.1 Gbp
Number of Scaffolds 410,604
Mean scaffold length 6,775 Kbp
Median length 139 bp
Quality stats:
Ns percentage 10.72% (r/ genome)
Ns percentage 11.94% (r/ scaffolds)
Scaffolds cover 89.74% of the genome
Gap percentage 10.26%
NG50 2.9 Mbp
NG80 535.3 Kbp
NG90 - bp
Build 7.1 scaffolds from ftp://ftp.xenbase.org/pub/Genomics/JGI/Xenla7.1/

## Slide 5
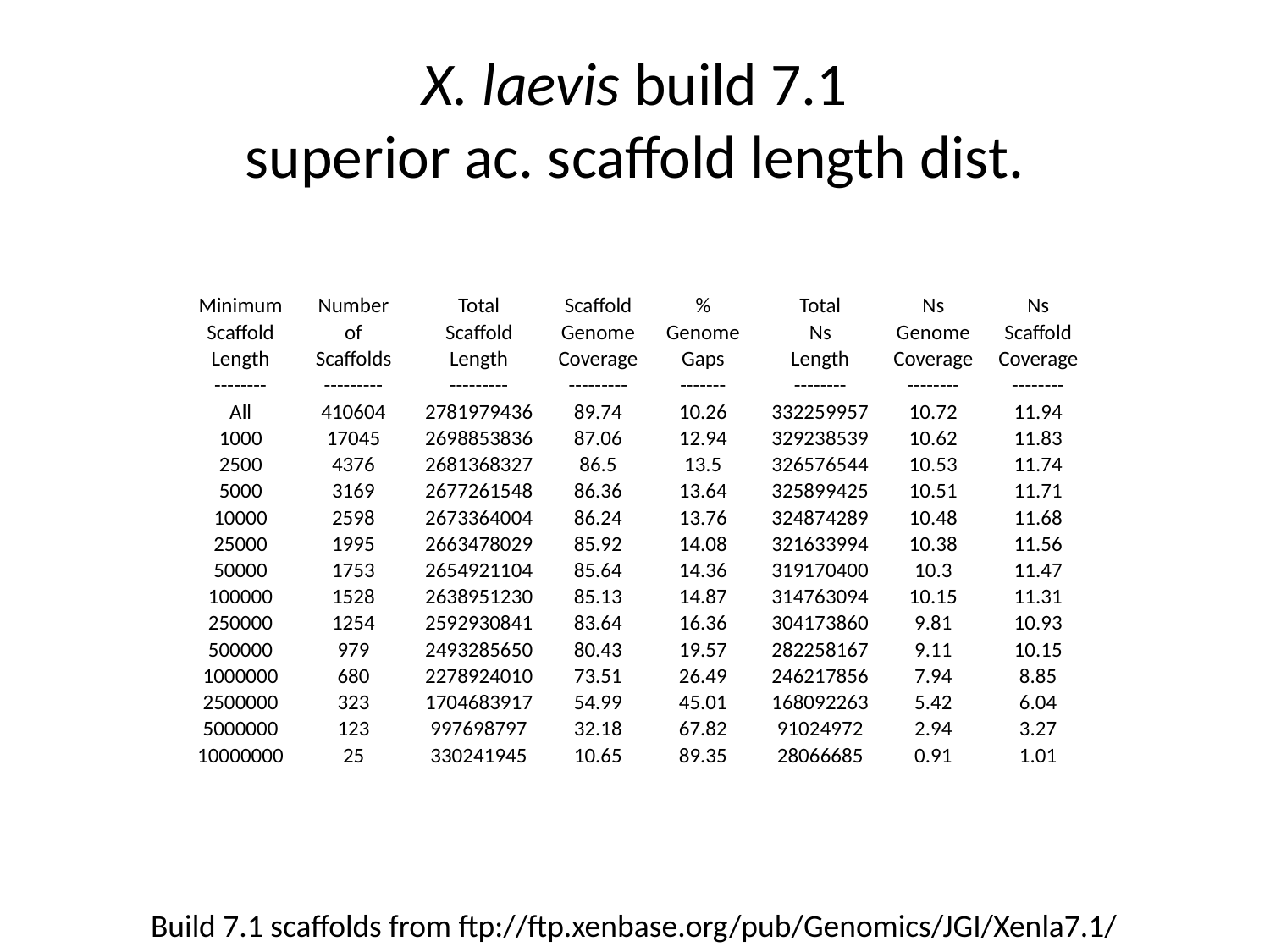

# X. laevis build 7.1 superior ac. scaffold length dist.
| Minimum | Number | Total | Scaffold | % | Total | Ns | Ns |
| --- | --- | --- | --- | --- | --- | --- | --- |
| Scaffold | of | Scaffold | Genome | Genome | Ns | Genome | Scaffold |
| Length | Scaffolds | Length | Coverage | Gaps | Length | Coverage | Coverage |
| -------- | --------- | --------- | --------- | ------- | -------- | -------- | -------- |
| All | 410604 | 2781979436 | 89.74 | 10.26 | 332259957 | 10.72 | 11.94 |
| 1000 | 17045 | 2698853836 | 87.06 | 12.94 | 329238539 | 10.62 | 11.83 |
| 2500 | 4376 | 2681368327 | 86.5 | 13.5 | 326576544 | 10.53 | 11.74 |
| 5000 | 3169 | 2677261548 | 86.36 | 13.64 | 325899425 | 10.51 | 11.71 |
| 10000 | 2598 | 2673364004 | 86.24 | 13.76 | 324874289 | 10.48 | 11.68 |
| 25000 | 1995 | 2663478029 | 85.92 | 14.08 | 321633994 | 10.38 | 11.56 |
| 50000 | 1753 | 2654921104 | 85.64 | 14.36 | 319170400 | 10.3 | 11.47 |
| 100000 | 1528 | 2638951230 | 85.13 | 14.87 | 314763094 | 10.15 | 11.31 |
| 250000 | 1254 | 2592930841 | 83.64 | 16.36 | 304173860 | 9.81 | 10.93 |
| 500000 | 979 | 2493285650 | 80.43 | 19.57 | 282258167 | 9.11 | 10.15 |
| 1000000 | 680 | 2278924010 | 73.51 | 26.49 | 246217856 | 7.94 | 8.85 |
| 2500000 | 323 | 1704683917 | 54.99 | 45.01 | 168092263 | 5.42 | 6.04 |
| 5000000 | 123 | 997698797 | 32.18 | 67.82 | 91024972 | 2.94 | 3.27 |
| 10000000 | 25 | 330241945 | 10.65 | 89.35 | 28066685 | 0.91 | 1.01 |
Build 7.1 scaffolds from ftp://ftp.xenbase.org/pub/Genomics/JGI/Xenla7.1/

## Slide 6
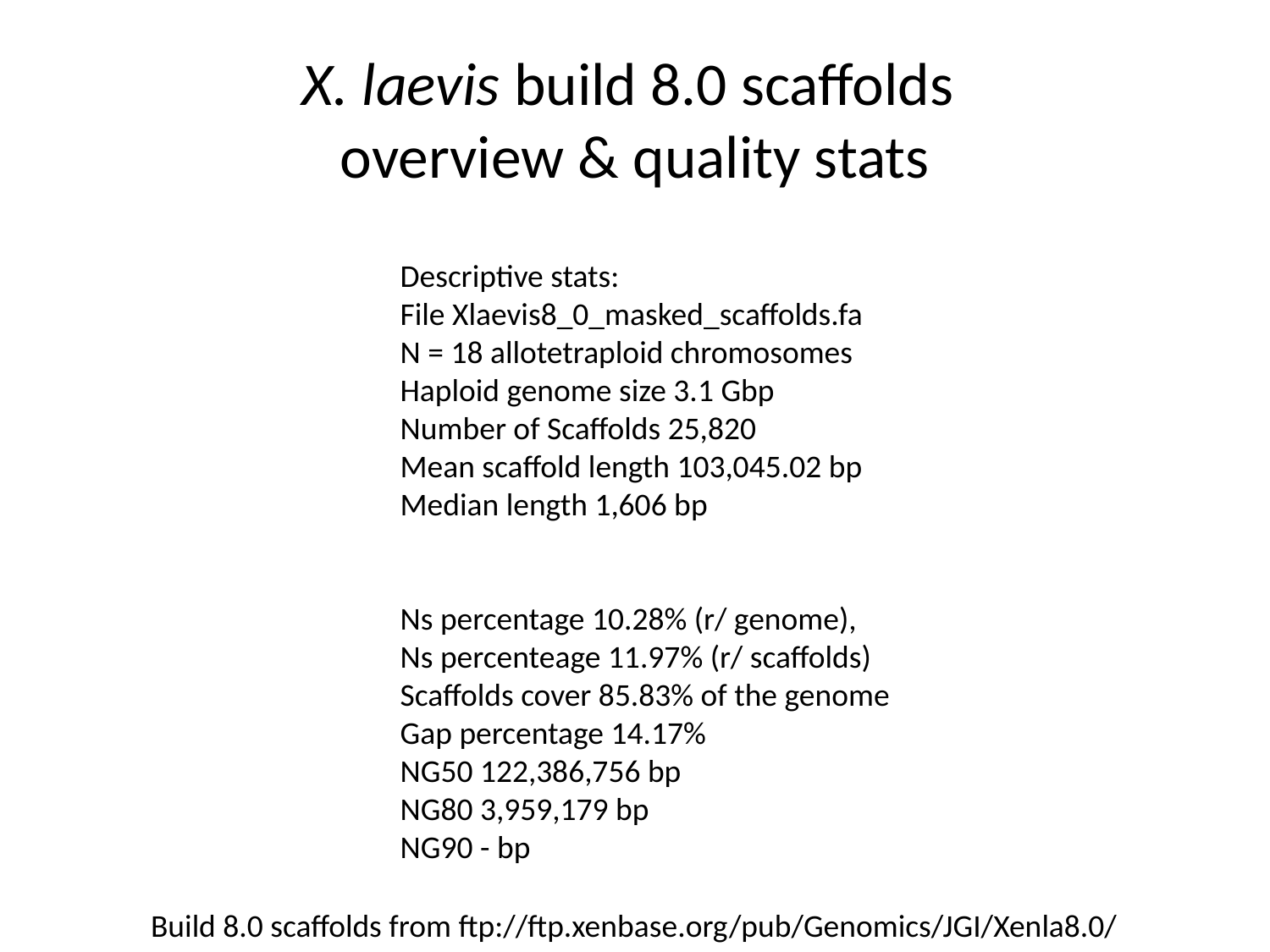

# X. laevis build 8.0 scaffolds overview & quality stats
Descriptive stats:
File Xlaevis8_0_masked_scaffolds.fa
N = 18 allotetraploid chromosomes
Haploid genome size 3.1 Gbp
Number of Scaffolds 25,820
Mean scaffold length 103,045.02 bp
Median length 1,606 bp
Ns percentage 10.28% (r/ genome),
Ns percenteage 11.97% (r/ scaffolds)
Scaffolds cover 85.83% of the genome
Gap percentage 14.17%
NG50 122,386,756 bp
NG80 3,959,179 bp
NG90 - bp
Build 8.0 scaffolds from ftp://ftp.xenbase.org/pub/Genomics/JGI/Xenla8.0/

## Slide 7
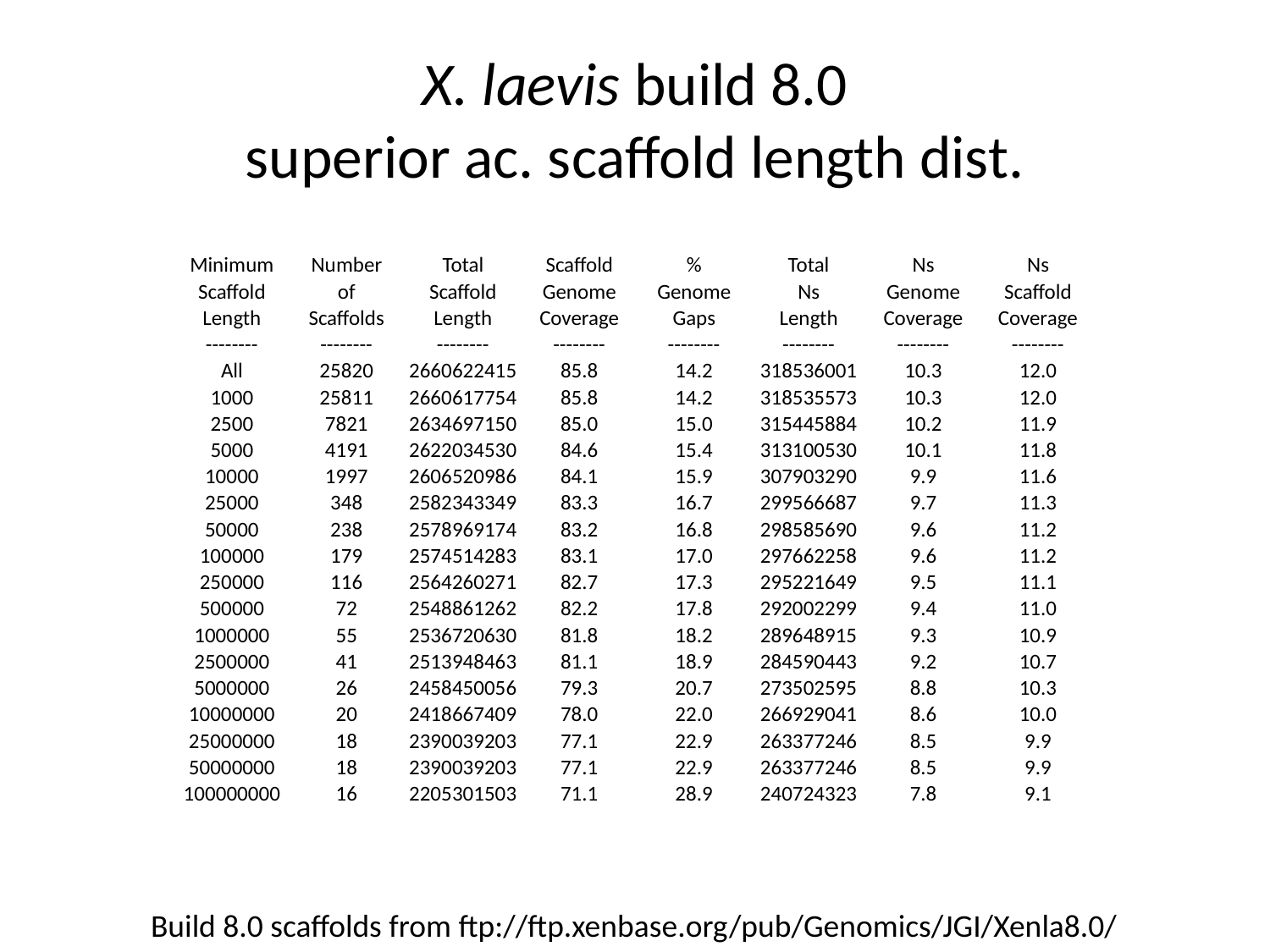

# X. laevis build 8.0 superior ac. scaffold length dist.
| Minimum | Number | Total | Scaffold | % | Total | Ns | Ns |
| --- | --- | --- | --- | --- | --- | --- | --- |
| Scaffold | of | Scaffold | Genome | Genome | Ns | Genome | Scaffold |
| Length | Scaffolds | Length | Coverage | Gaps | Length | Coverage | Coverage |
| -------- | -------- | -------- | -------- | -------- | -------- | -------- | -------- |
| All | 25820 | 2660622415 | 85.8 | 14.2 | 318536001 | 10.3 | 12.0 |
| 1000 | 25811 | 2660617754 | 85.8 | 14.2 | 318535573 | 10.3 | 12.0 |
| 2500 | 7821 | 2634697150 | 85.0 | 15.0 | 315445884 | 10.2 | 11.9 |
| 5000 | 4191 | 2622034530 | 84.6 | 15.4 | 313100530 | 10.1 | 11.8 |
| 10000 | 1997 | 2606520986 | 84.1 | 15.9 | 307903290 | 9.9 | 11.6 |
| 25000 | 348 | 2582343349 | 83.3 | 16.7 | 299566687 | 9.7 | 11.3 |
| 50000 | 238 | 2578969174 | 83.2 | 16.8 | 298585690 | 9.6 | 11.2 |
| 100000 | 179 | 2574514283 | 83.1 | 17.0 | 297662258 | 9.6 | 11.2 |
| 250000 | 116 | 2564260271 | 82.7 | 17.3 | 295221649 | 9.5 | 11.1 |
| 500000 | 72 | 2548861262 | 82.2 | 17.8 | 292002299 | 9.4 | 11.0 |
| 1000000 | 55 | 2536720630 | 81.8 | 18.2 | 289648915 | 9.3 | 10.9 |
| 2500000 | 41 | 2513948463 | 81.1 | 18.9 | 284590443 | 9.2 | 10.7 |
| 5000000 | 26 | 2458450056 | 79.3 | 20.7 | 273502595 | 8.8 | 10.3 |
| 10000000 | 20 | 2418667409 | 78.0 | 22.0 | 266929041 | 8.6 | 10.0 |
| 25000000 | 18 | 2390039203 | 77.1 | 22.9 | 263377246 | 8.5 | 9.9 |
| 50000000 | 18 | 2390039203 | 77.1 | 22.9 | 263377246 | 8.5 | 9.9 |
| 100000000 | 16 | 2205301503 | 71.1 | 28.9 | 240724323 | 7.8 | 9.1 |
Build 8.0 scaffolds from ftp://ftp.xenbase.org/pub/Genomics/JGI/Xenla8.0/

## Slide 8
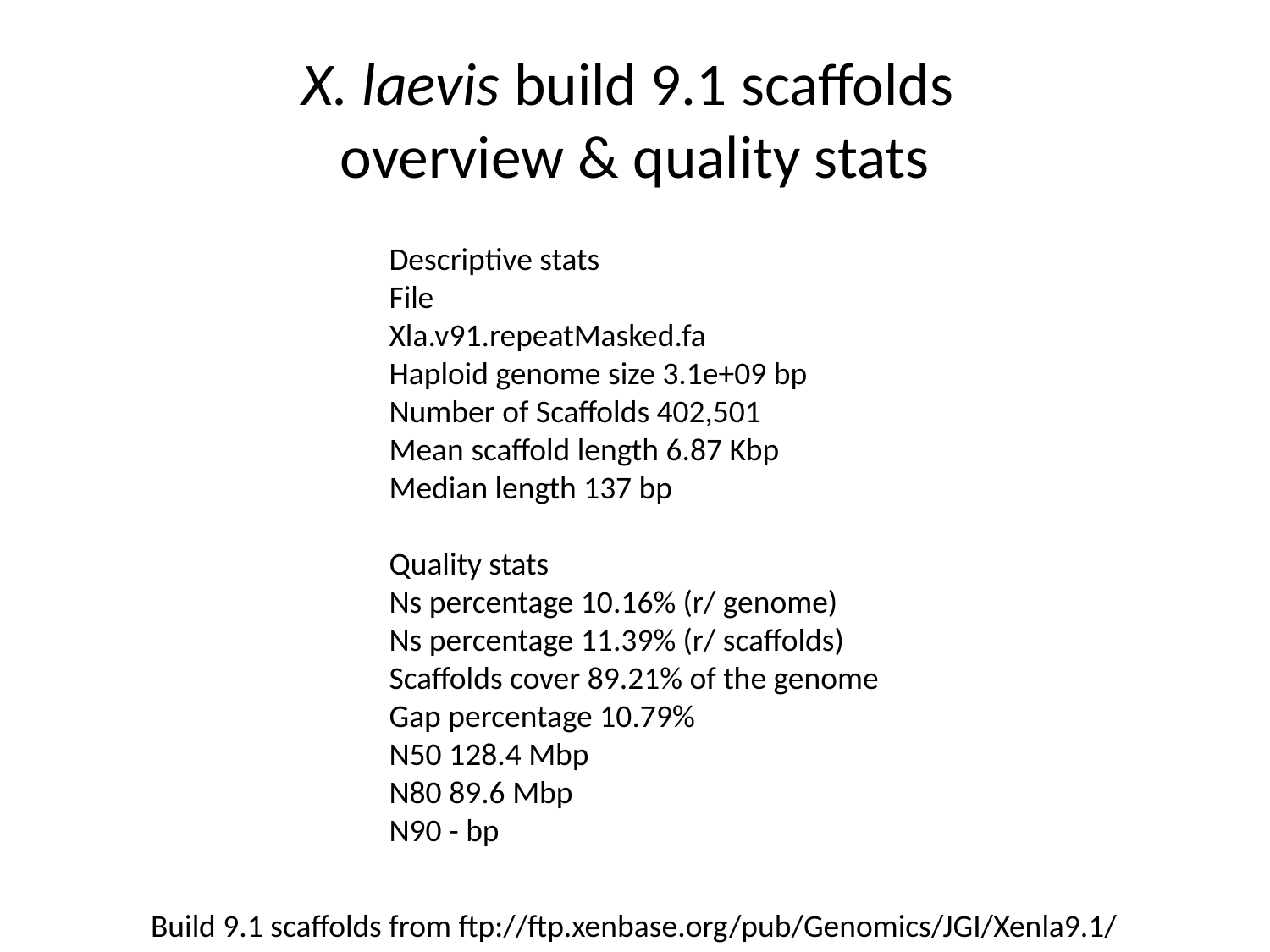

# X. laevis build 9.1 scaffolds overview & quality stats
Descriptive stats
File
Xla.v91.repeatMasked.fa
Haploid genome size 3.1e+09 bp
Number of Scaffolds 402,501
Mean scaffold length 6.87 Kbp
Median length 137 bp
Quality stats
Ns percentage 10.16% (r/ genome)
Ns percentage 11.39% (r/ scaffolds)
Scaffolds cover 89.21% of the genome
Gap percentage 10.79%
N50 128.4 Mbp
N80 89.6 Mbp
N90 - bp
Build 9.1 scaffolds from ftp://ftp.xenbase.org/pub/Genomics/JGI/Xenla9.1/

## Slide 9
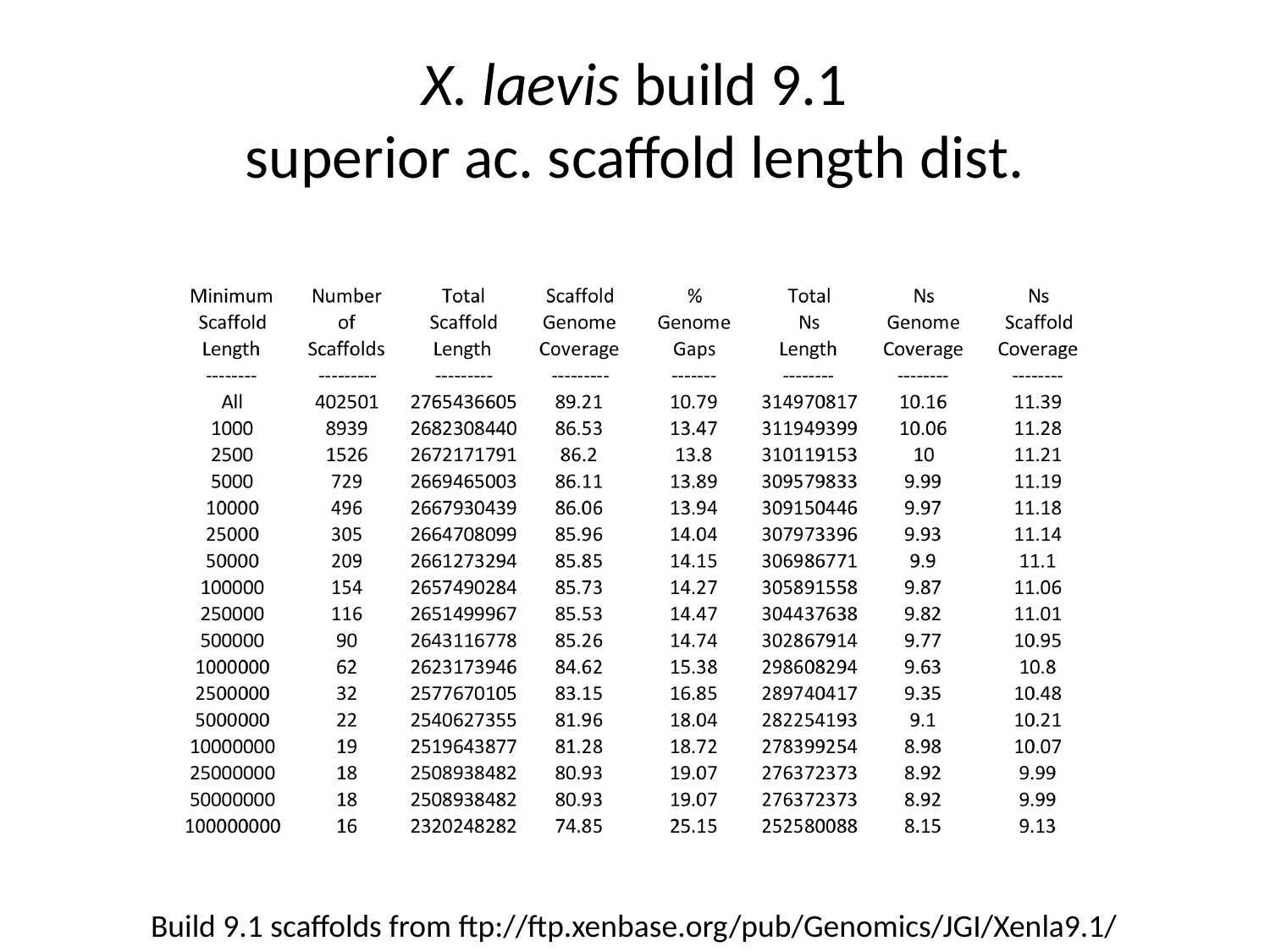

# X. laevis build 9.1 superior ac. scaffold length dist.
Build 9.1 scaffolds from ftp://ftp.xenbase.org/pub/Genomics/JGI/Xenla9.1/

## Slide 10
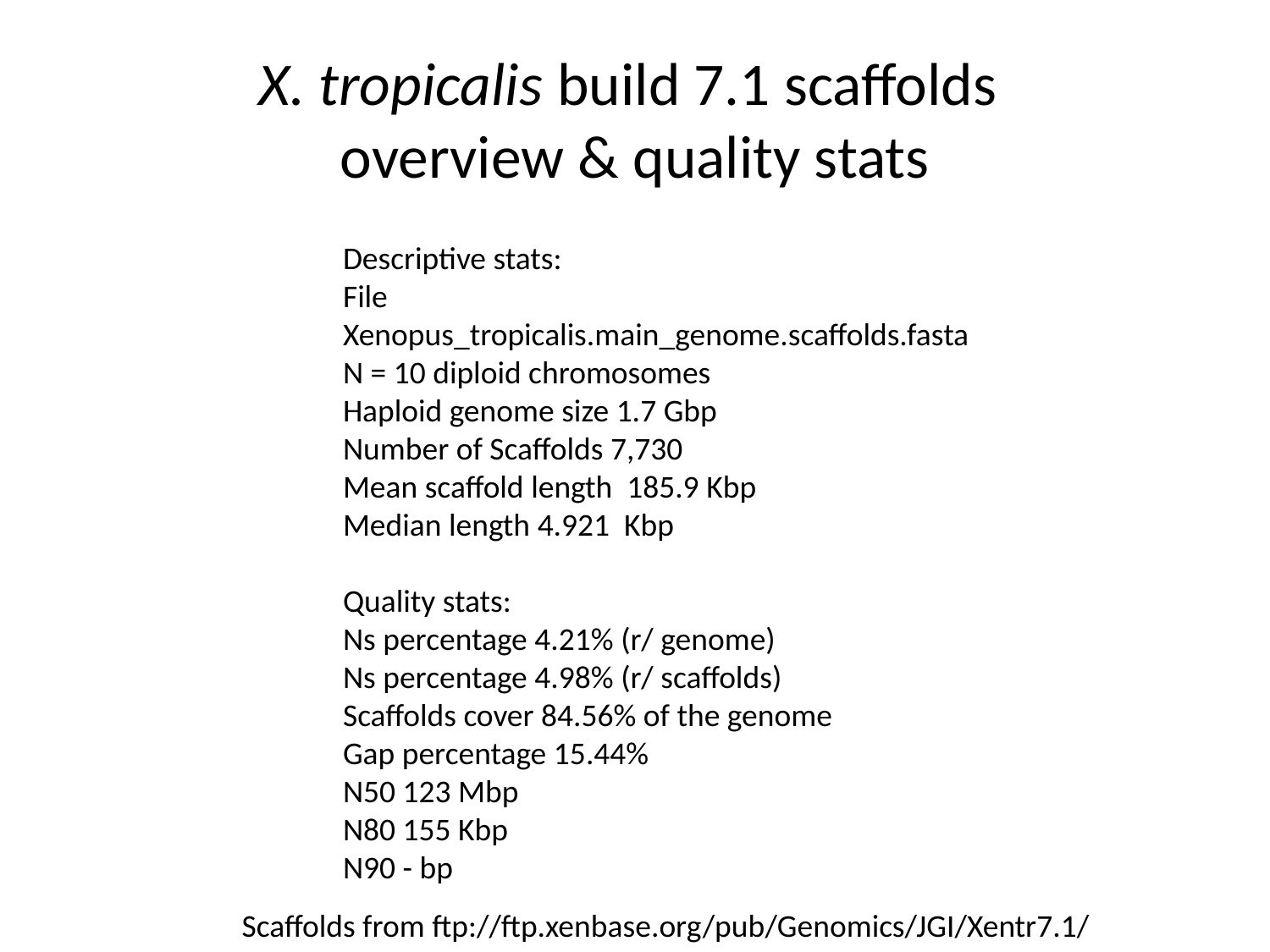

# X. tropicalis build 7.1 scaffolds overview & quality stats
Descriptive stats:
File Xenopus_tropicalis.main_genome.scaffolds.fasta
N = 10 diploid chromosomes
Haploid genome size 1.7 Gbp
Number of Scaffolds 7,730
Mean scaffold length 185.9 Kbp
Median length 4.921 Kbp
Quality stats:
Ns percentage 4.21% (r/ genome)
Ns percentage 4.98% (r/ scaffolds)
Scaffolds cover 84.56% of the genome
Gap percentage 15.44%
N50 123 Mbp
N80 155 Kbp
N90 - bp
Scaffolds from ftp://ftp.xenbase.org/pub/Genomics/JGI/Xentr7.1/

## Slide 11
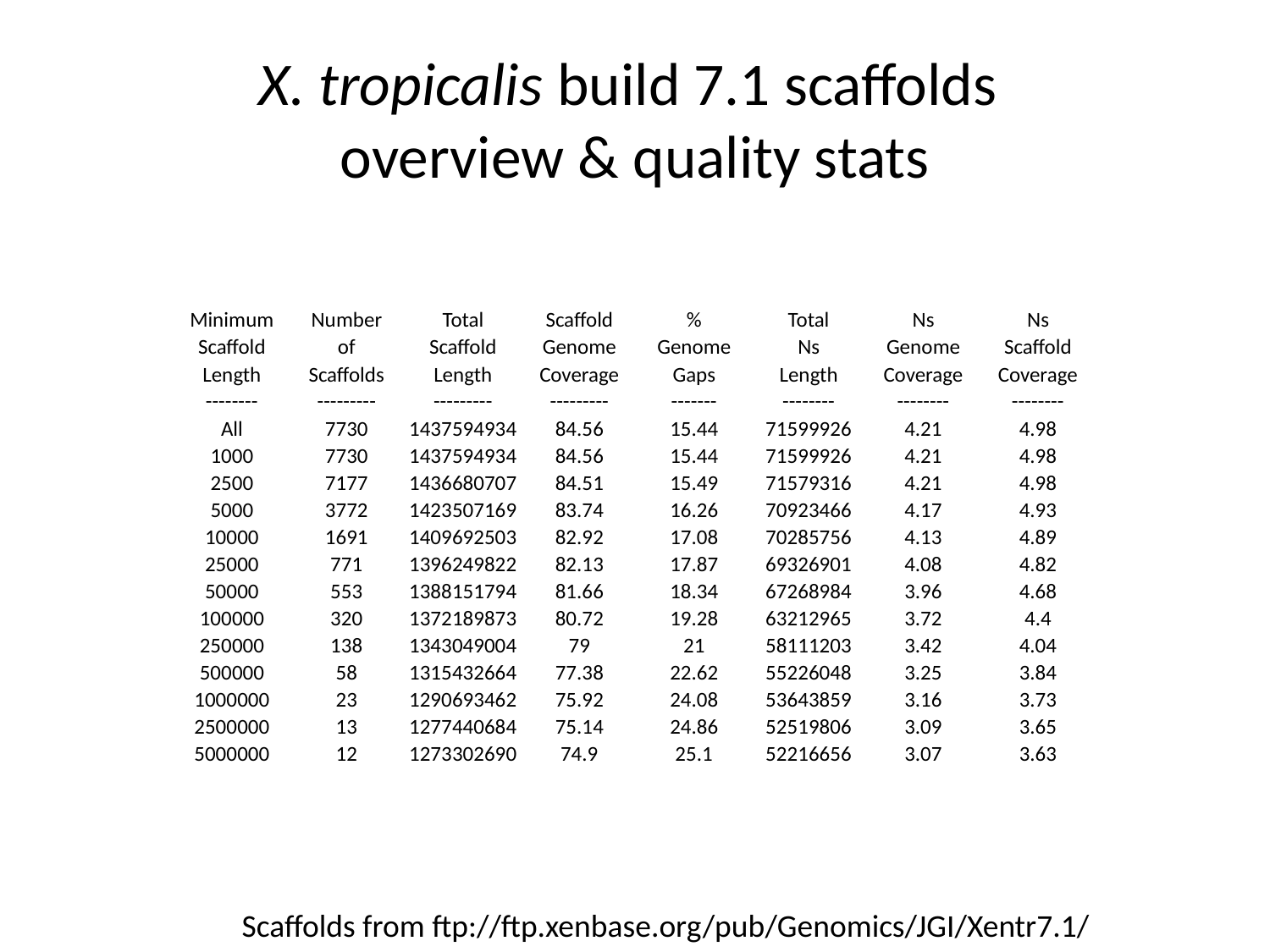

# X. tropicalis build 7.1 scaffolds overview & quality stats
| Minimum | Number | Total | Scaffold | % | Total | Ns | Ns |
| --- | --- | --- | --- | --- | --- | --- | --- |
| Scaffold | of | Scaffold | Genome | Genome | Ns | Genome | Scaffold |
| Length | Scaffolds | Length | Coverage | Gaps | Length | Coverage | Coverage |
| -------- | --------- | --------- | --------- | ------- | -------- | -------- | -------- |
| All | 7730 | 1437594934 | 84.56 | 15.44 | 71599926 | 4.21 | 4.98 |
| 1000 | 7730 | 1437594934 | 84.56 | 15.44 | 71599926 | 4.21 | 4.98 |
| 2500 | 7177 | 1436680707 | 84.51 | 15.49 | 71579316 | 4.21 | 4.98 |
| 5000 | 3772 | 1423507169 | 83.74 | 16.26 | 70923466 | 4.17 | 4.93 |
| 10000 | 1691 | 1409692503 | 82.92 | 17.08 | 70285756 | 4.13 | 4.89 |
| 25000 | 771 | 1396249822 | 82.13 | 17.87 | 69326901 | 4.08 | 4.82 |
| 50000 | 553 | 1388151794 | 81.66 | 18.34 | 67268984 | 3.96 | 4.68 |
| 100000 | 320 | 1372189873 | 80.72 | 19.28 | 63212965 | 3.72 | 4.4 |
| 250000 | 138 | 1343049004 | 79 | 21 | 58111203 | 3.42 | 4.04 |
| 500000 | 58 | 1315432664 | 77.38 | 22.62 | 55226048 | 3.25 | 3.84 |
| 1000000 | 23 | 1290693462 | 75.92 | 24.08 | 53643859 | 3.16 | 3.73 |
| 2500000 | 13 | 1277440684 | 75.14 | 24.86 | 52519806 | 3.09 | 3.65 |
| 5000000 | 12 | 1273302690 | 74.9 | 25.1 | 52216656 | 3.07 | 3.63 |
Scaffolds from ftp://ftp.xenbase.org/pub/Genomics/JGI/Xentr7.1/

## Slide 12
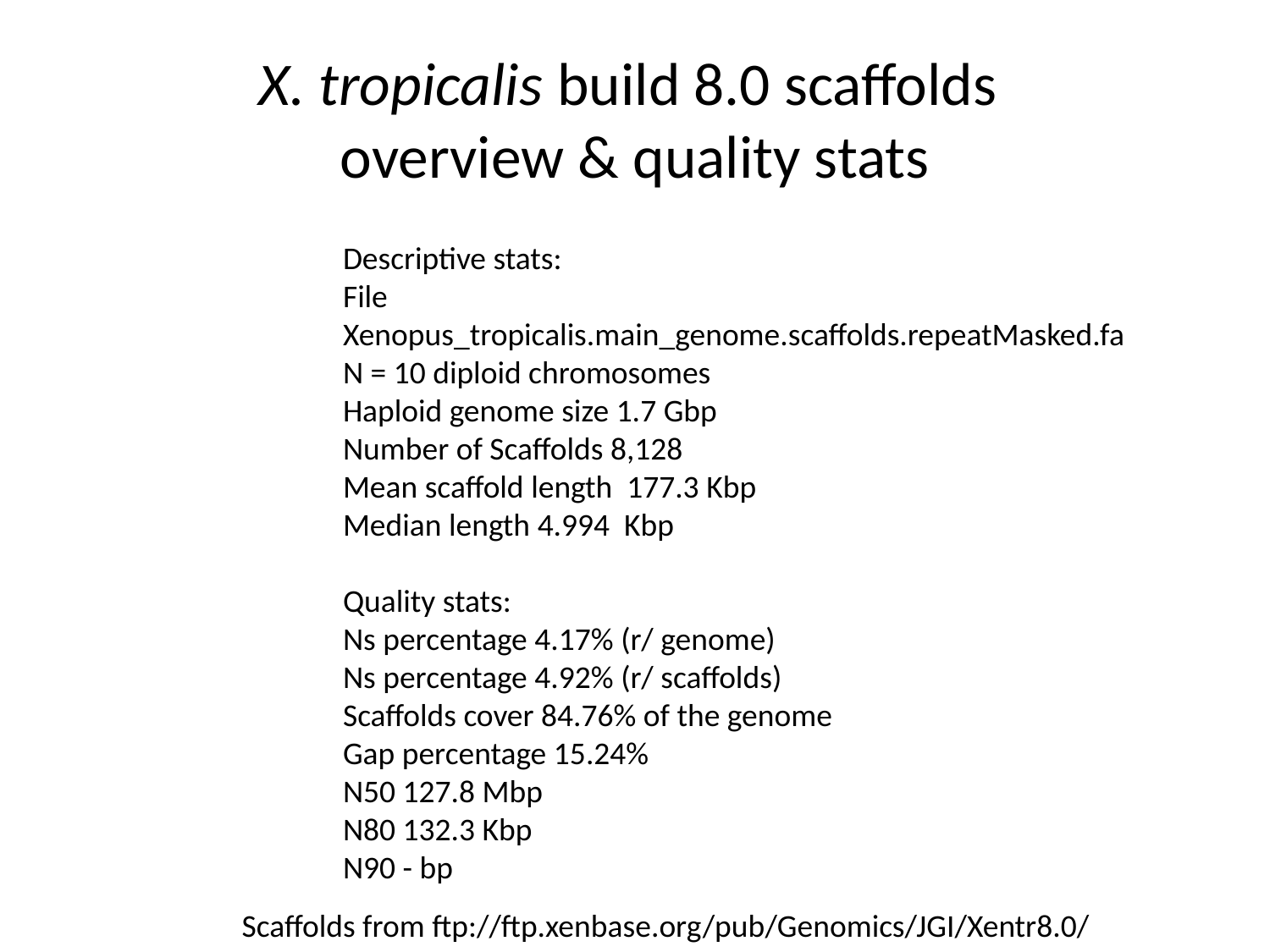

# X. tropicalis build 8.0 scaffolds overview & quality stats
Descriptive stats:
File Xenopus_tropicalis.main_genome.scaffolds.repeatMasked.fa
N = 10 diploid chromosomes
Haploid genome size 1.7 Gbp
Number of Scaffolds 8,128
Mean scaffold length 177.3 Kbp
Median length 4.994 Kbp
Quality stats:
Ns percentage 4.17% (r/ genome)
Ns percentage 4.92% (r/ scaffolds)
Scaffolds cover 84.76% of the genome
Gap percentage 15.24%
N50 127.8 Mbp
N80 132.3 Kbp
N90 - bp
Scaffolds from ftp://ftp.xenbase.org/pub/Genomics/JGI/Xentr8.0/

## Slide 13
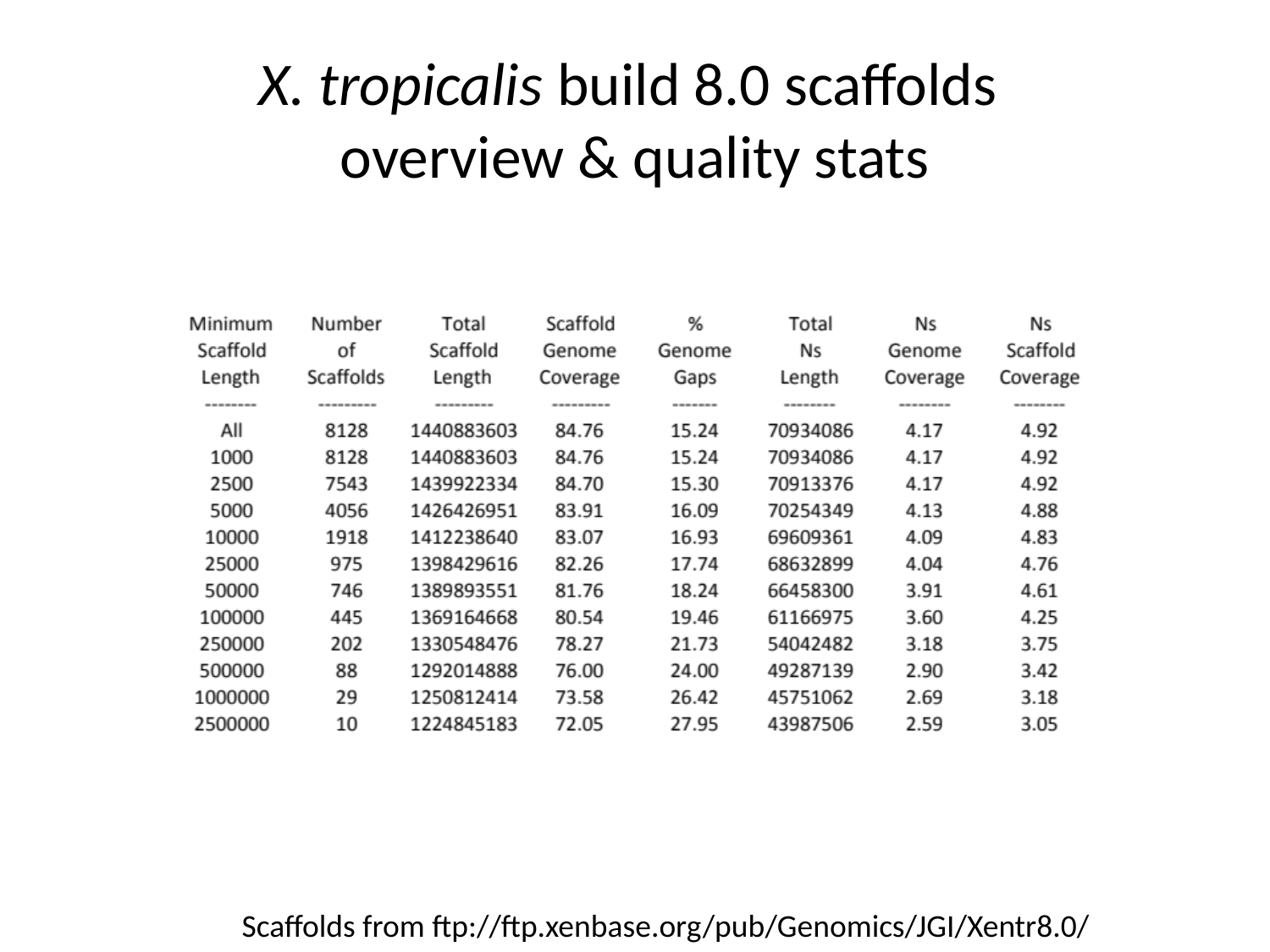

# X. tropicalis build 8.0 scaffolds overview & quality stats
Scaffolds from ftp://ftp.xenbase.org/pub/Genomics/JGI/Xentr8.0/

## Slide 14
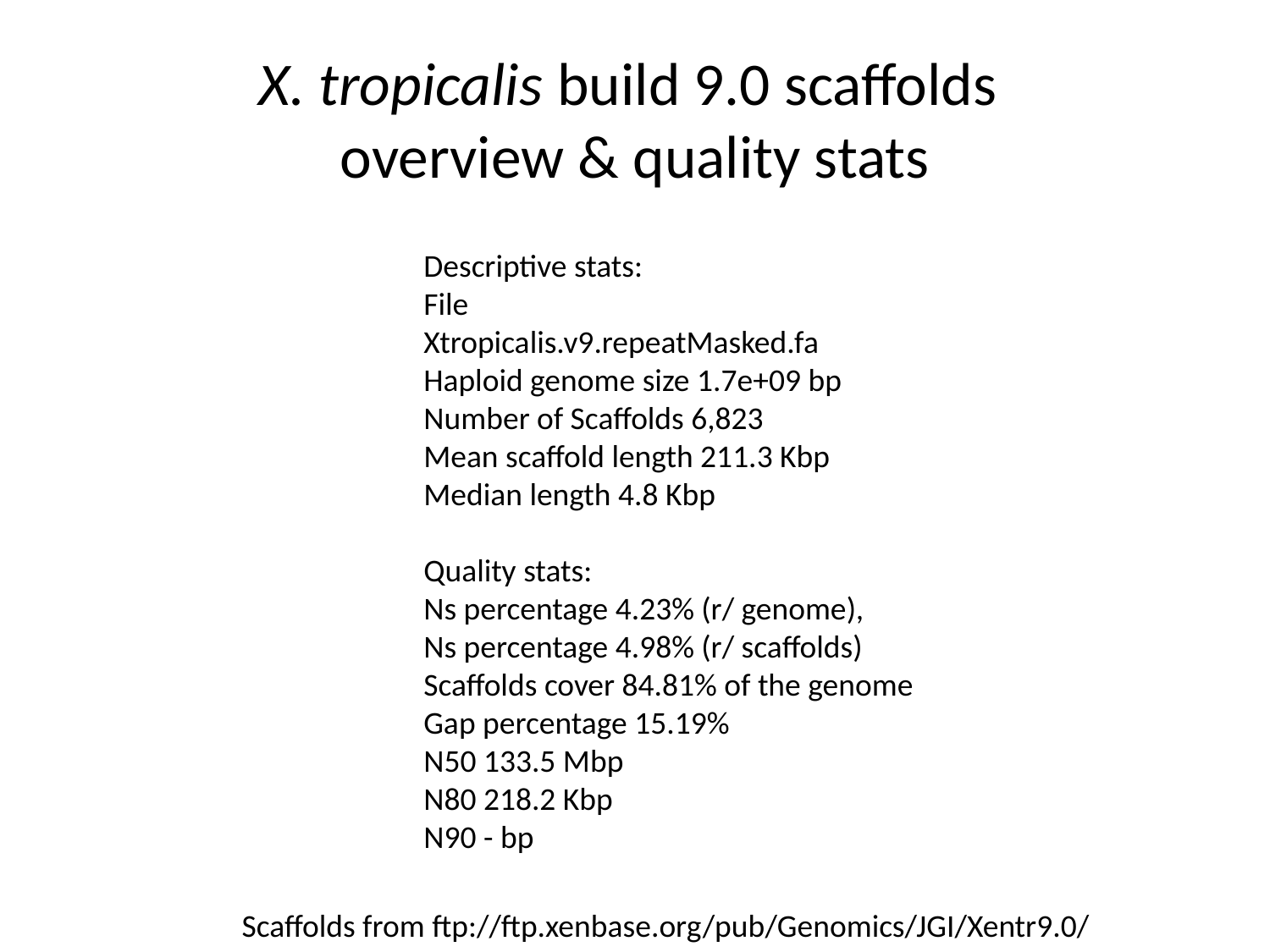

# X. tropicalis build 9.0 scaffolds overview & quality stats
Descriptive stats:
File
Xtropicalis.v9.repeatMasked.fa
Haploid genome size 1.7e+09 bp
Number of Scaffolds 6,823
Mean scaffold length 211.3 Kbp
Median length 4.8 Kbp
Quality stats:
Ns percentage 4.23% (r/ genome),
Ns percentage 4.98% (r/ scaffolds)
Scaffolds cover 84.81% of the genome
Gap percentage 15.19%
N50 133.5 Mbp
N80 218.2 Kbp
N90 - bp
Scaffolds from ftp://ftp.xenbase.org/pub/Genomics/JGI/Xentr9.0/

## Slide 15
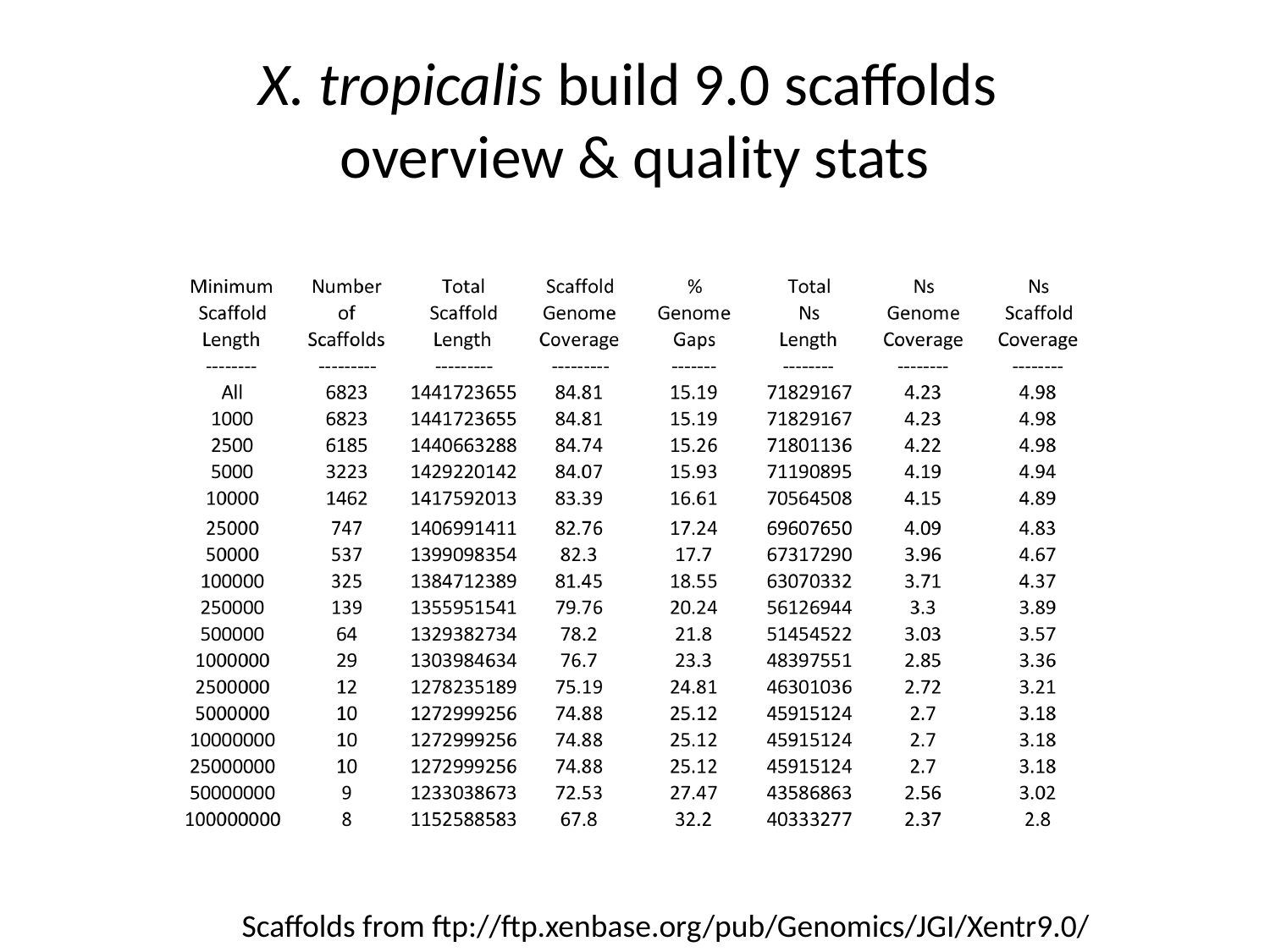

# X. tropicalis build 9.0 scaffolds overview & quality stats
Scaffolds from ftp://ftp.xenbase.org/pub/Genomics/JGI/Xentr9.0/
